# Dimensional Analysis over Finite Holographic Substrate

**DOI:** 10.3390/e28101122

**Published:** 2026-10-14

**Authors:** Yosef Akhtman

**Affiliations:** 1 Gamma Earth Sàrl, 1162 St-Prex, Switzerland; ya@gamma.earth; 2 Faculty of Space Technologies, AGH University of Krakow, 30-059 Krakow, Poland

**Keywords:** dimensional analysis, finite ring cosmology, physical units, Planck units, physical constants, modular domains, quantum mechanics, mass, gravitational constant, speed of light

## Abstract

The paper derives the fundamental physical units in a universe defined over a finite holographic substrate: a finite totality whose capacity bounds the state count of every embedded observer. The units are anchored at the substrate’s four horizons: the Planck length, momentum, time and energy. The constants *c*, *ℏ*, *G*, $k_{B}$ follow uniquely from this quartet, over realisations stated with their falsifiers: *c* the ratio across the two Fourier pairs, *ℏ* the product within each conjugate pair, $k_{B}$ the mixed product, *G* the normalisation to the totality. The quartet admits one cancellation identity and no further independent relation; on an admissible substrate the constants are exact residues read as unit-face magnitudes. Beneath the quartet, dimensional analysis is a graded modular domain algebra on the framed shell, recovering the classical calculus exactly inside the sub-capacity window. The domain of *ℏ* is the unit flag; its torsion-free surrogate is the classical mass dimension, temperature inherits the acceleration domain, and the flag cancels in every count-valued comparison. Two predictions are staked: a resolution floor on single coherent registration and the exact constancy of the constants’ dimensionless ratios. Every theorem of the paper is numerically verified and formalized in Lean 4.

## 1. Introduction

Dimensional analysis is one of the oldest and most robust consistency principles in physics. Before a physical equation is tested, solved, or interpreted, it must satisfy dimensional homogeneity: only quantities of the same physical kind can be added, while products and ratios move between physical kinds according to fixed algebraic rules. This requirement is often treated as bookkeeping. In practice, it is closer to a grammar of physical law: it determines which expressions can carry physical meaning before any particular dynamics is specified.

The classical formulation of this grammar is the theory of dimensions and similarity. Rayleigh’s discussion of similitude and Buckingham’s formulation of the Pi theorem made explicit that physically meaningful laws are constrained before their numerical coefficients are known [1,2]. If a quantity has dimension $L^{r}T^{s}M^{u}\cdots$, the exponents record how it transforms under changes of unit scale, and dimensionless monomials are the invariant combinations of this exponent map. Later treatments by Bridgman and Barenblatt developed the same idea into a systematic method for scaling and similarity analysis [3,4]. This viewpoint belongs to the broader symmetry tradition: physical content is identified with what remains invariant under an admissible class of transformations [5,6,7]. Modern algebraic treatments of quantity calculus formalise the same structure exactly: Raposo’s group-graded quantity calculus and Jonsson’s quantity spaces take the dimension group free abelian [8,9]; Zapata-Carratalá’s dimensioned algebra admits arbitrary abelian dimension structures in its general formalism, while its physical instantiations remain torsion-free [10]; Alymov develops the quantity field as a fibrewise, partially additive structure [11]; and a recent gauge-theoretic reading derives the quantity calculus from unit covariance [12]. The present construction differs precisely where finiteness bites: the exponent lattice here is bicyclic, with the two distinct periods p and p−1, and it carries torsion, an order-four unit flag that no free dimension group and no torsion-free instantiation contains.

The finite state count itself has independent grounding in physics. The black-hole entropy bound [13], dimensional reduction at the Planck scale [14], the holographic principle [15], and the de Sitter horizon entropy [16] place a finite bound on the number of physical states behind any horizon, and Wheeler’s it-from-bit programme reads every physical quantity as a registered answer to a finite question [17]. Those programmes derive the count as a constraint on continuum theories. The present framework inverts the reading: the finite count is the substrate itself. The totality carries a finite capacity S, its cardinality follows as Ω=4S+1, and a bounded observer is an embedded shell whose chart fits inside the count, $p^{2}<\Omega$: the encoding condition. The minimal exact realisation of the holographic principle is constructed and machine-verified in [18]: the complete objectless quantum dynamics runs on the pair (13,233), the total state is the register on the holographic encoding of the observer’s sky, and the construction is a model existence proof for the finite substrate. The present paper works one level below the dynamics: on a holographic substrate the fundamental units are the observer-facing readings of the one capacity, and dimensional analysis is the grammar of what a bounded observer can register across its own horizon.

The same tradition left open the status of the physical constants. Planck’s construction of natural units showed that *c*, *G*, *ℏ*, and $k_{B}$ pass between conventional units and natural scale assignments [19], and the long discussion that followed asks how many constants are fundamental and which are conversion conventions [20,21,22,23]. The present paper gives that question a definite answer within the Finite Ring Cosmology (FRC) framework [24,25], where the fundamental physical units are shown to be the four horizons of the finite holographic substrate: the Planck length $\ell_{P}$, Planck momentum $p_{P}$, Planck time $t_{P}$, and Planck energy $E_{P}$. The four fundamental constants then follow uniquely from this quartet. The speed of light is the ratio across the two Fourier pairs of the domain square, the Planck constant the product within each conjugate pair, the Boltzmann constant the mixed product (itself the derived monomial ℏ/c in magnitude, read in the Planck-anchored chart), and the gravitational constant the normalisation of the quartet to the totality. Exactly three of the four are independent: the quartet admits exactly one internal cancellation identity, and no further algebraically independent relation exists within its lattice.

The mathematical setting is finite algebra rather than continuum analysis. Finite fields provide exact cyclic additive and multiplicative structures [26]; finite geometries and finite Fourier analysis carry geometry and harmonic structure without an actual continuum [27,28,29]; finite-dimensional quantum and phase-space constructions use finite fields as exact phase coordinates [30,31,32]. In FRC, shell residues are coordinate primitives; they acquire physical roles only when a frame assigns them, and the assignment is relational [33,34]. We work with a framed prime shell given capacity-first: the primary datum is the capacity κ, the shell’s information budget, and the shell cardinality follows as p=4κ+1, prime, for the framed shell $F_{p}\left(\tau ;0,1,g\right)$. The construction makes the phase cycle’s order 4κ divisible by four automatically, so the quarter-turn subgroup is present on every shell: the *symmetry-complete* case of the framework’s earlier papers, here automatic and therefore named only this once. The frame data carry four distinct roles, kept separate throughout: the chronon τ is the Subject’s temporal origin (its “now”), 0 the spatial origin (its “here”), 1 the unit, and g the multiplier of the Subject’s own drive x↦gx. Section 2 places this shell inside the full architecture of the framework: the Carrier (the finite totality), the Subject (the bounded observer), and the Object (an observed system), each with its own register of residues.

The paper then has two layers. The lower layer is the observer’s grammar: the two charts of the framed shell generate finite modular unit domains ${\left[L\right]}^{r}{\left[T\right]}^{s}$, $\left(r,s\right)\in D_{p}=C_{p}\times C_{p-1}$, with fibrewise addition and a neutral-domain invariant criterion, the graded algebra $A_{p}$ whose three governing statements (the grading law, the fibrewise addition rule, and the neutral-domain criterion, Theorems 2–4) constitute the precise sense of the word grammar throughout. Classical dimensional analysis is recovered exactly inside the sub-capacity window, at crossing degree at most one (Theorem 10, Corollary 9); the crossing bound is not cosmetic, since beyond it the flag sector saturates modulo four. The upper layer is the substrate anchor: the four representation domains of the shell each carry a Carrier-scale horizon, the quartet above, and the constants are derived from it (Section 6).

The status of the claims is drawn explicitly row by row (see Appendix A). Claims are tagged: T an algebraic theorem, proved in the text; D a definition; R a realisation, a row stating what a physical term denotes in the substrate, exact as a statement about domains, constitutive rather than interpretive, defended by its registered consequences and killed by its explicit falsifier [18]; I an import; Ω an Ω-hard residue, fixed by the totality and inaccessible to any bounded observer. The algebraic layer is deliberately elementary: a bicyclic graded group algebra over $C_{p}\times C_{p-1}$ and a bounded-window injectivity argument; no new theorem of finite or algebraic number theory is claimed. The claimed contributions are three. First, the bicyclic domain proposal: the two domain-generator periods attached to the additive and multiplicative charts of one framed shell. The time-exponent period p−1 follows from the character-grade identification of the time exponent, a constitutive declaration exhibited by the reframing covariance, from which the period is then forced; the space-exponent refinement to p is declared as the meridian’s native coordinate period (Remark 3). The bicyclic proposal is two declarations of different downstream status, and it is stated as such.

The classical dimension group cannot express this structure, since the dimension symbols of the Bridgman tradition form a free abelian, hence torsion-free, group. Second, the domain of the Planck constant as the unit flag $i_{D}={\left[T\right]}^{\kappa }$: the generator of the unique order-four subgroup the domain lattice itself carries, at the capacity exponent, $i_{D}^{4}=1$, inexpressible within every sub-capacity window H<κ. The flag marks the crossing between the observer’s counts (frequency, wavenumber) and the substrate-anchored measures (energy, momentum), and the classical three-generator system arises by adjoining a free primitive, the mass dimension, as the flag’s torsion-free surrogate. The crossing’s order-four premise is argued, not declared: the flag is the record of the fractional-Fourier quarter that the chart duality forgets (Proposition 3). Third, the quartet theorem of Section 6: two anchored horizons and one crossing quantum determine the four constants uniquely. Each constant’s residue pair is the unique admissible solution of a bounded-height defining relation, and no further algebraically independent relation is available within the quartet lattice; the Carrier instantiation enters as the existence certificate, not as the subject. The analysis is conducted where the observer lives, on the unit face.

The proposed formalism is unrelated to the idea of “natural units”, where some of the fundamental constants are set to 1 by fiat. The present construction sets no constant to 1; it identifies the geometric objects the constants connect. The numerical values of the constants change with the choice of conventional units; the horizons and modular domains they represent do not.

The paper is organized as follows. Section 2 fixes the Carrier–Subject–Object architecture, the frame roles, and the shell charts. Section 3 separates residue phase labels from framed-complex labels. Section 4 defines the modular unit-domain group and homogeneous quantity algebra and proves the homogeneity and invariant criteria. Section 5 establishes the four representation domains, the chart duality, and the unit flag. Section 6 states the quartet and proves the unique derivation of the constants. Section 7 derives the mechanical and gravitational domains as corollaries. Section 8 explains finite periodicity, gives examples, and recovers classical bookkeeping. Section 9 summarizes the result and its scope. Appendix A is the predicate ledger: forty-eight rows in nine blocks, one predicate per row, each tagged and sourced. Its theorem rows link their Lean 4 (v4.34.0) declarations and, where a witness is named, their Python (v3.13.16) checks. Block P states the two predictions with their falsifiers and present confrontations; block X restates, in four rows, what classical bookkeeping postulates and the construction derives.

## 2. The Carrier, the Subject Shell, and the Registers

This section fixes the architecture the dimensional analysis lives in: the finite totality, the bounded observer, and the observed system, each with its own register of residues, and the frame charts and scale readings placed over them.

**Definition 1**  (The Carrier)**.** *The finite, complete totality is the Carrier C: a timeless torsor of *Ω* attribute-free points, whose cardinality is unique because the totality is one, and is its only absolute characteristic. Its reframings x↦ax+b are automorphisms, so what a frame marks as *0*, *1*, −1 is observer frame data, never absolute structure; primality is the characteristic’s algebraic face (a self-contained totality admits no proper substrate beneath its chart). The Carrier’s primary datum is its capacity S, the de Sitter entropy, with the cardinality its first derivative, Ω=4S+1. A Carrier is admissible when *Ω* is prime, S is even (equivalently Ω≡1(mod8), the two-way residue class) and S≡1(mod3); the three clauses are adopted as a standing definition (Remark 1), their origin the framework’s instantiation layer. A frame coordinatizes C as the prime field $F_{\Omega }$; the field is the frame’s chart of the Carrier, not the Carrier itself.*

**Definition 2**  (The Subject and its frame)**.** *A Subject S is a bounded observer: an embedded prime shell of capacity κ (the shell’s* information budget*, a dimensionless size, the quarter of the phase cycle, with no temporal content), with the cardinality derived, and frame data *

$$F_{p}\left(\tau ;0,1,g\right),\;p=4\kappa +1\;\mathrm{prime},\;p^{2}<\Omega .$$

*The phase cycle has order 4κ by construction, so the quarter-turn subgroup is automatic on every shell. A Subject is admissible when κ>1, the shell strictly containing its quarter-turn core: $F_{5}$ is that core itself (4κ=4), κ=2 gives 9, composite, and the least admissible Subject is p=13; the condition $p^{2}<\Omega$ is the pair’s coherence clause, the encoding condition of Remark 1. The four frame data carry four distinct roles. The chronon τ is the Subject’s* temporal origin *(which orbit point is “now”), exactly parallel to 0, the spatial origin (“here”), and 1, the unit; it is an origin, not a size and not a unit, assigned by the observer and shared by none. The generator g is the multiplier of the Subject’s own drive x↦gx, invariant under every reframing (conjugating the drive by x↦ax+b returns a drive with the same g); the shell constrains only ord(g)∣4κ, and the framed shell takes its drive primitive, ord(g)=4κ, the full cycle. From its frame the shell derives its unitless structural residues: the oriented quarter-turn $i=-g^{\kappa }=g^{-\kappa }$ with $i^{2}=-1$, the half-period π=2κ with $g^{\pi }=-1$ and 2π≡−1, and the exponential unit $e=g^{\;i}$. A residue used as an exponent is read through the frame’s canonical representative: the framed shell carries the canonical coordinate window {0,1,…,p−1}⊂Z, fixed by its origin and unit, and a residue enters an exponent as that representative, so $e=g^{\;i}$ is g raised to the integer representative of i (at p=13, g=2: i=5, $e=2^{5}=6$). Exponents combine modulo 4κ while the coordinate window is p-periodic, and the two periods differ, so the representative reading is part of the frame datum rather than an implicit convention; the verification suite records its representative convention and checks it inert wherever an identity is representative-sensitive.*

**Remark 1**  (The holographic substrate)**.** *The two definitions together are the paper’s reading of the holographic principle. The capacity S is the de Sitter entropy in the sense of the horizon-entropy literature (I): the finite count of physical states behind the observer’s horizon [13,15,16]. The exact content here is the congruence Ω=4S+1, not an asymptotic: in Planck cells the area law A=4S reads A=Ω−1; the origin is the one point no boundary chart registers. The congruence, the primality of *Ω*, and the admissibility clauses of Definitions 1 and 2 are the framework’s realisation of the bound (R), never derived from the area law: the law motivates the finite count, the realisation instantiates it. The framework likewise reads the dimensionless A/4 number as the count itself, a capacity, not the logarithm of a state multiplicity; that reading is part of the same realisation. Both faces of the law are observed (I): the measured cosmological constant fixes the de Sitter horizon, whose A/4 entropy evaluates to $\left(2.6\pm 0.3\right)\times 10^{122}\;k_{B}$ [35], and horizon-area non-decrease is supported observationally on GW150914 [36]. The admissibility $p^{2}<\Omega$ is the encoding condition: the Subject’s framed-complex label space $C_{p}=F_{p}\times F_{p}$ (Definition 7), of $p^{2}$ ordered-pair labels, fits inside the Carrier count, so a bounded observer is an encoding on the substrate rather than a part-whole inclusion. A finite totality whose capacity bounds every embedded observer’s chart in this sense is a finite holographic substrate; the minimal admissible pair (13,233), minimal under the complete admissibility predicate of Definitions 1 and 2, carries the minimal exact realisation of the principle, machine-verified as a model existence proof [18], and Section 6 reads the same capacity at the unit face. Minimality is by exhaustive scan (Appendix A, row E9): without the mod3 clause (13,193) would precede the pair, and without κ>1 the degenerate p=5 pairs would. The instantiation layer of the minimal pair strengthens the parity clause to S≡2(mod4), jointly Ω≡41(mod48) [18]; this paper adopts the even clause, and the declaration is deliberate: the two forms agree on (13,233) (S=58≡2(mod4)), the scan returns the pair under either, and they diverge only at S≡0(mod4), where the framework’s laboratory Carrier, S=602,140 (Section 6), satisfies the adopted triple and not the strengthened form. Which parity form is canonical is an instantiation-layer question outside this paper’s scope, and nothing here depends on it.*

**Remark 2**  (The scale of the shells)**.** *The encoding condition bounds the Subject’s cardinality and does not prescribe it: a bounded observer is any admissible shell whose label space fits the count, and the paper fixes no physical p. The bound $\sqrt{\Omega }$, of the order of $10^{61}$ for the de Sitter capacity read on the chart, is the saturating-Subject scale (Remark 19), not the shell of any embedded observer. Every theorem below holds on every admissible shell and consumes no numeral: it is proved once for all κ; the toy shell $F_{13}$ is its arithmetic display, the minimal pair the framework’s machine-verified model existence [18], and the public suite repeats checks on larger shells and on both instantiations it runs, the minimal pair and the laboratory Carrier. An observer never reaches its modulus inside a coherent window: a registered count is read through the frame’s canonical representative below p (Definition 2), and the framework’s registrability bound on coherent counts is $\sqrt{p}$ (its exponent reading the coherence window of Remark 19), quadratically below the period. And the wrap itself is cheap: a prime of the order of $10^{61}$ has some two hundred bits, and exact arithmetic modulo such a prime costs a few dozen machine operations per field multiplication, the everyday scale of elliptic-curve cryptography [37]; the cost grows polynomially with the bit length of p, never with p.*

**Definition 3**  (The Object)**.** *An observed system O is registered relative to the Subject’s frame of reference, every entry a Subject residue, (t;q,k,v): the relative time t (the Object’s synchronisation datum relative to the Subject’s now: observing a star, the Subject’s now is τ, and the star observed now was at t relative to τ), the position q, the winding k (its scale mark; mass is winding rate), and the rate v (its registered step multiplier). One relative datum per frame datum: t↔τ, q↔0, k↔1, v↔g.*

**Registers and hardness.** Each of the three carries its own register of residues, and hardness is the register signature. The Carrier register is $\left\{S,\Omega ,c,\hbar ,G,k_{B}\right\}$, all Ω-hard Carrier residues, generator-free in the capacity S alone: G=2S the half-cycle (2G≡−1), $c^{2}=2S+1=2^{-1}$, $\hbar^{2}\equiv -1$ (the quarter-turn pair), $k_{B}^{2}\equiv -2$, with the *h*-form h=2πℏ≡−ℏ and the pair linkage $\left\{\pm k_{B}\right\}\left\{\pm c\right\}=\left\{\pm \hbar \right\}$. The Subject register is {p,κ,i,π,e}: all p-hard Subject residues, the frame (τ;0,1,g) the Subject’s own. Cross-register value coincidences are one residue read in two registers, a frame’s reading, never an identity. These registers are the two tiers the domain analysis below separates: the observer’s counts are Subject-register quantities, the unitful measures are anchored at the Carrier, and the crossing between the tiers is the unit flag of Section 5.

**The two charts.** One shell carries two cyclic coordinate actions. The additive chart $\left(F_{p},+\right)$, of cardinality p, supplies the meridian or space-like frame chart relative to the chosen origin 0: if the observer assigns the scale label “meter” to the unit 1, a coordinate $x\in F_{p}$ receives an *x*-meter spatial reading in that frame; assigning “millimeter” changes the reading, not the shell. The multiplicative chart $F_{p}^{\times }$, of order 4κ=p−1, supplies the phase-action chart relative to the drive: the powers of g enumerate the nonzero phase cycle. Time enters through the drive (each application of x↦gx is one chronon step, the frame’s temporal unit), so the temporal scale assignment attaches to the drive’s step, while the chronon τ itself is the origin the steps are counted from. A change of drive presentation is written $g^{\prime }=g^{\epsilon }$, $\epsilon \in Z_{p-1}^{\times }$.

**Figure 1:**
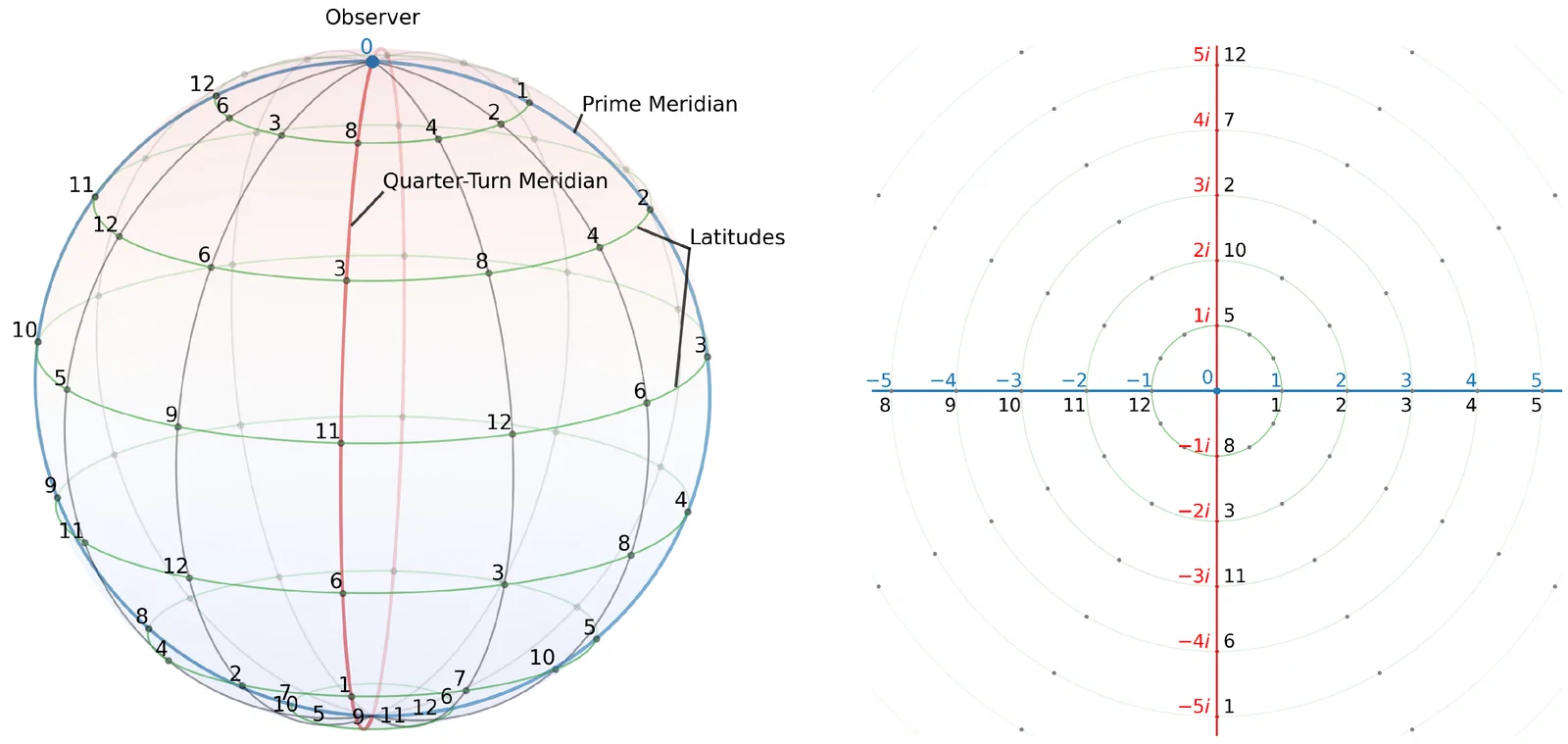
The framed holographic shell $F_{13}\left(\tau ;0,1,g\right)$ of capacity κ=3, cardinality p=4κ+1=13, chronon τ (the observer’s “now”), position 0 (the observer’s “here”), scale 1, primitive generator g=2, oriented quarter-turn $i=-g^{\kappa }=5$, exponential unit $e=g^{i}=6$, and half-period π=2κ=6. (**Left**): the orbital 2-sphere: the observer origin 0 at the north pole, the additive prime meridian (blue), the quarter-turn meridian (red), and the multiplicative latitudes (green). (**Right**): the framed-complex Euclidean chart a+bi over $F_{13}$: the prime meridian as the real axis, the quarter-turn meridian as the imaginary axis (1i=5), and the latitudes as norm circles.

Figure 1 shows these data for p=13, κ=3. The left panel is the orbital 2-sphere of the shell [24]: the observer origin at the north pole, the additive prime meridian and the quarter-turn meridian crossing it, and the multiplicative latitudes of the drive. The right panel reads the same shell as the framed-complex Euclidean chart a+bi: the prime meridian as the real axis, the quarter-turn meridian as the imaginary axis, and the latitudes as norm circles. Every mark, including the quarter-turn $i=-g^{\kappa }$, is a finite-field residue of the one shell.

**Figure 2:**
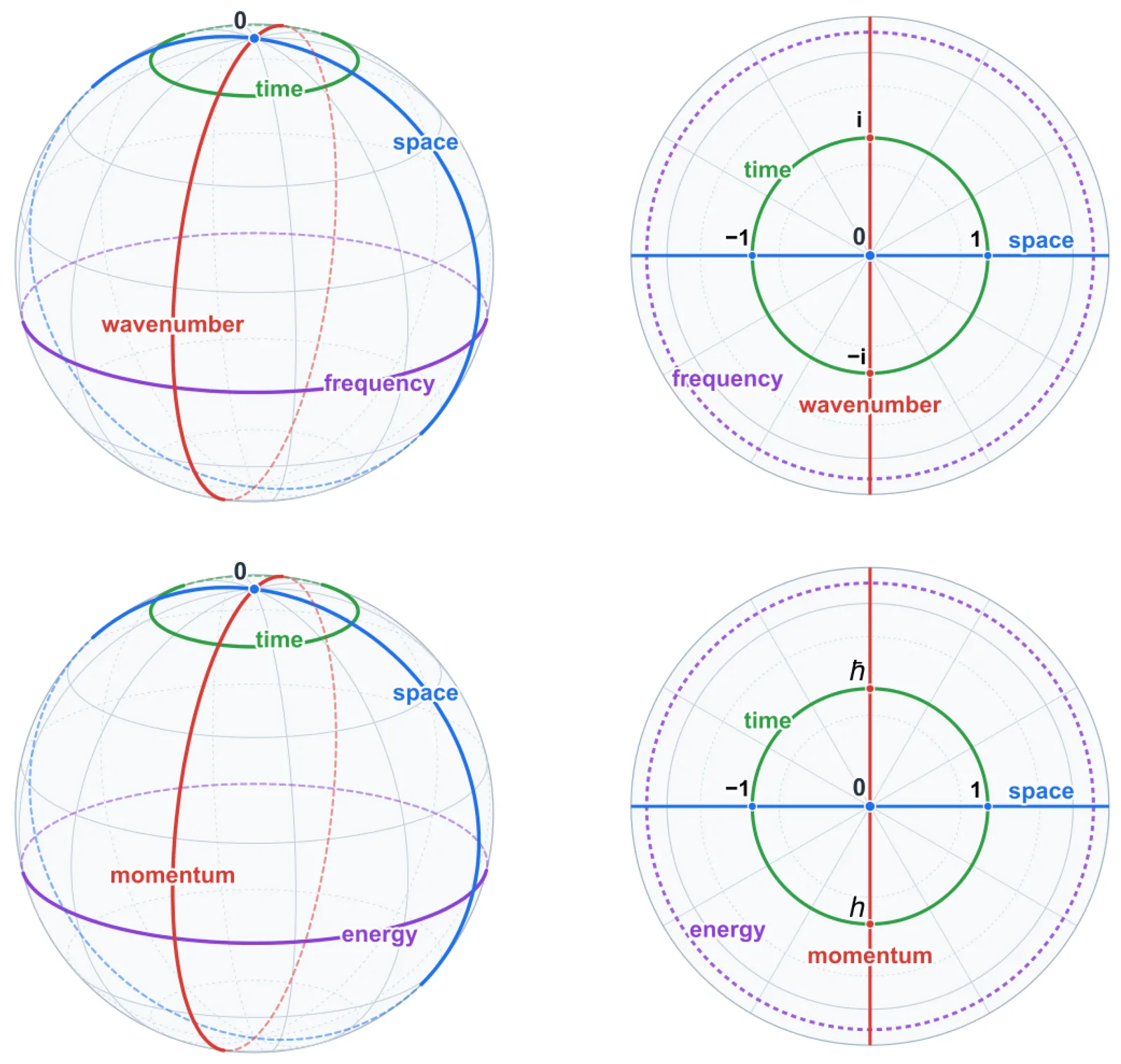
The four representation domains a framed holographic shell, in both Subject and Carrier registers. The Subject register (**top**): space (the prime meridian, blue) and wavenumber (red) are the figure transverse additive-Fourier pair; time (green) and frequency (purple) are the longitudinal multiplicative-Fourier pair, the drive running along time. (**Left**): the orbital sphere with the observer origin 0 at the pole. (**Right**): the same data flattened into the observer’s chart, with the cardinal residues 0,±1,±i, all marks observer frame data, the flag-free counts of the four charts, unitless relative to the frame’s own anchors; the chart duality $\delta_{S}$ of Theorem 6 pairs each domain with its reciprocal. The Carrier register (**bottom**): the same domains with the dual charts named by their unitful readings, momentum and energy, anchored at the substrate. The primal charts, space and time, are identical across the rows, while each dual chart differs from its count by exactly the flag $i_{D}$ of Theorem 7, carried by the Planck constant: flag positions (0,0,1,1), Proposition 8. On the Carrier ring the quarter-turn role i is held by *ℏ*, and the opposite role by h=−ℏ: the {ℏ,h} pair. The cancellation identity $|k_{B}|\;c=\hbar$ drawn on the chart in its representative form (Corollary 3, Theorem 9).

Figure 2 assembles the full architecture on the same holographic shell: the two charts and their Fourier duals (the four representation domains) read in both registers, one row per register. It names the quantities whose domains the analysis below establishes: the observer’s counts (wavenumber, frequency) in the Subject register, and the unitful measures (momentum, energy) anchored at the Carrier, with the crossing between the rows carried by the Planck constant.

**Figure 3:**
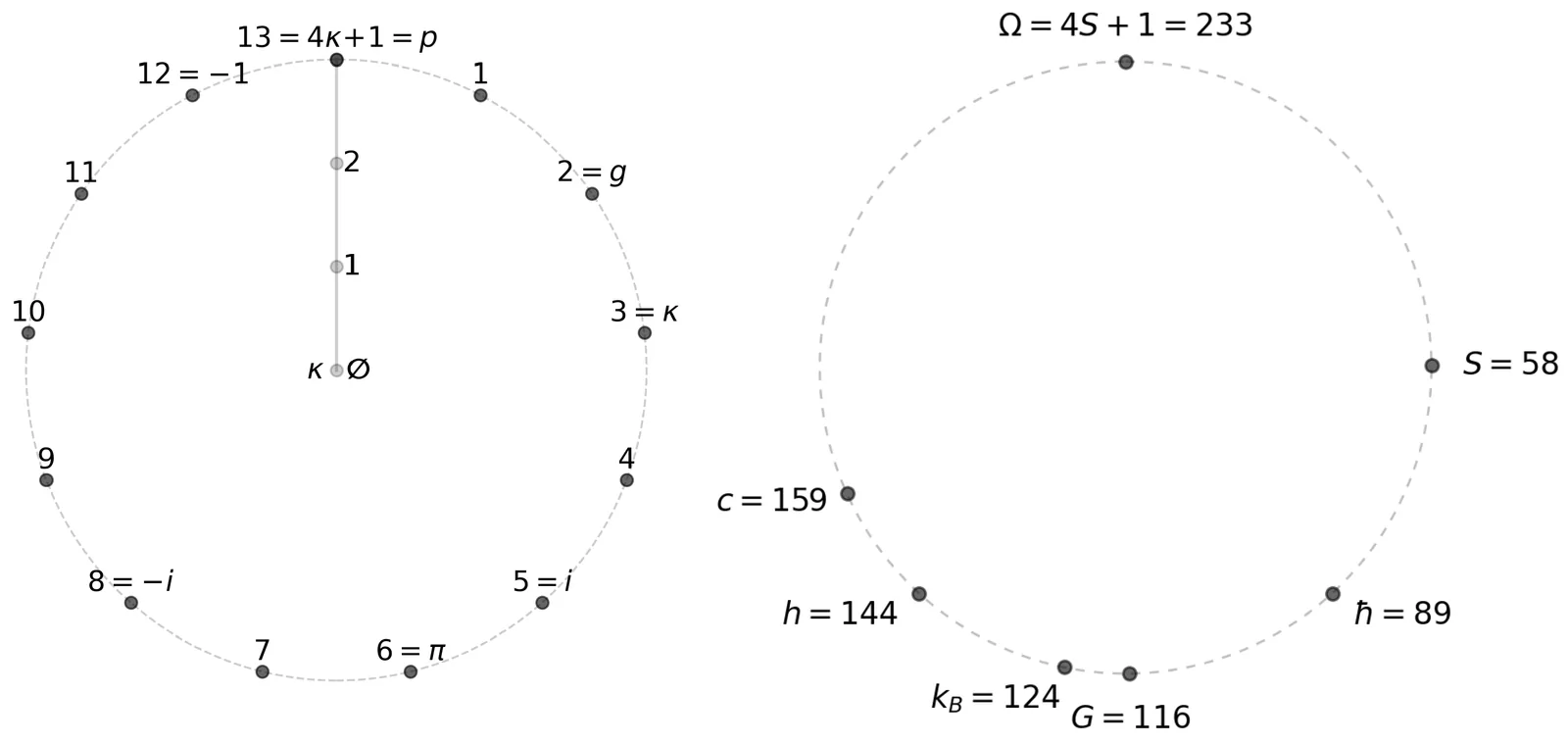
The two registers at the smallest admissible scale, (p,Ω)=(13,233). Subject (**left**): the shell $F_{13}$ a frame derives internally (generator g=2, capacity κ=3, quarter-turn i=5, half-period π=6, with −i=8, −1=12, and the wrap point p≡0 carrying its framed label (3;0)); the radial axis holds the shell’s information capacity. Carrier (**right**): Ω=233, capacity S=58, displaying the residue pairs whose unit-face magnitudes the observer registers as the physical constants (Theorem 9): G=2S=116 exact at the half-cycle; the root pair of −1 is {89,144}, the {ℏ,h}*h*-form pair, both members marked; *c* and $k_{B}$ shown by the suite’s representatives 159 ($c^{2}\equiv 2^{-1}$) and 124 ($k_{B}^{2}\equiv -2$), pair partners 74 and 109. Marks are representative-convention data; the pairs are the Carrier facts.

Figure 3 closes the architecture at the smallest admissible scale: the two registers whole, the observer’s holographic shell beside the presented minimal Carrier, (p,Ω)=(13,233), the pair minimal under the admissibility predicate of Definitions 1 and 2. The left ring is what a Subject frame derives internally; the right ring displays the residue pairs whose unit-face magnitudes the observer registers as the physical constants (Section 6).

**Definition 4**  (Unit-free coordinate actions)**.** *The additive meridian action $\left(F_{p},+\right)$ and multiplicative phase action $F_{p}^{\times }$ are unit-free coordinate actions. A physical unit is a scale assignment to a frame chart of these actions. A measure is a coordinate reading relative to the assigned scale.*

**Definition 5**  (Typed modular reading)**.** *A physical-interface reading is not a bare residue. It is a coefficient together with the domain label in which it is read. The two periods type the label exponents, not the coefficient: the space-like exponent is counted in the order-p factor and the time-like exponent in the order-(p−1) factor of the domain lattice (Section 4), while the coefficient of every homogeneous fibre lies in $F_{p}$. Readings carrying different domain labels cannot be added unless an explicit admissible conversion has first been specified.*

**Proposition 1**  (Scale assignment preserves shell cardinality)**.** *Changing the physical scale assigned to the additive meridian or multiplicative phase chart does not change the finite shell. In particular,*

$$\mathrm{card}\left(F_{p}\right)=p,\;\mathrm{card}\left(F_{p}^{\times }\right)=p-1$$

*are invariant under such scale assignments.*

**Proof.**  A scale assignment labels the reading of coordinates already present in $F_{p}$ or $F_{p}^{\times }$. It does not alter the underlying finite set, its addition, its multiplication, or its group order. Hence the cardinalities remain p and p−1. □

The affine part of the frame is expressed by transformations $x^{\prime }=mx+a$ with $a\in F_{p}$, $m\in F_{p}^{\times }$: *a* changes origin and *m* changes spatial scale, the frame logic of the framed finite-field algebra. The phase part is expressed by reindexing $g^{\prime }=g^{\epsilon }$. The dimensional analysis developed below uses these frame charts and requires no additional coordinate ontology.

## 3. Residue Phase Labels and Framed-Complex Labels

Quantum notation uses an imaginary unit to express phase evolution. In FRC the relevant quarter-turn is already present as a finite-field residue [24]. The purpose of this section is to separate two label-level formalisms for the same shell phase geometry. Throughout, the upright i denotes the shell residue, while the italic *i* denotes the imaginary-unit symbol of continuum notation.

The phase cycle has order 4κ by construction, hence a unique subgroup of order four. In a chosen clockwise frame this subgroup is written

$$Q_{p}=\left\{1,-i,-1,i\right\},\;i^{2}=-1.$$

If g is the chosen primitive generator, then $g^{\kappa }$ generates this order-four subgroup, and the established orientation convention writes $i=-g^{\kappa }=g^{-\kappa }$. The present paper uses only the fact that $g^{\kappa }$ is a cardinal quarter-turn whose square is −1.

There are two convenient ways to label this structure.

**Definition 6**  (Residue phase label)**.** *The residue phase label writes a phase as a power $g^{k}\in F_{p}^{\times }$. In this notation the cardinal packet is*

$$1,\;\;g^{\kappa },\;\;g^{2\kappa },\;\;g^{3\kappa },\;\;g^{4\kappa }=1.$$

**Definition 7**  (Framed-complex label)**.** *The framed-complex label space is the set of ordered pairs $C_{p}:=F_{p}\times F_{p}$, a pair (a,b) written a+bi (Figure 1, right), together with the evaluation map $\mathrm{ev}:C_{p}\to F_{p}$, (a,b)↦a+bi. The label space carries $p^{2}$ labels; evaluation is p-to-one, and the evaluated label is a finite-field residue already present in $F_{p}$, not an external imaginary extension. A framed-complex label is thus a chart datum, the ordered pair; its evaluation is the residue the chart denotes. The same quarter-turn structure recurs at totality scale: on the minimal Carrier of Figure 3 the root pair of −1 is the {ℏ,h} pair of Section 6.*

**Proposition 2**  (Quarter-turn label)**.** *In a framed prime shell of capacity κ, the element i is the finite shell quarter-turn label. Multiplication by i is the shell analogue of the quarter-turn represented by i in the continuum complex notation of quantum mechanics.*

**Proof.**  The group $F_{p}^{\times }$ is cyclic of order 4κ by construction, hence contains a unique subgroup of order four, generated by $g^{\kappa }$. Any generator i of that subgroup satisfies $i^{4}=1$ and $i^{2}\neq 1$. Since the unique element of order two in $F_{p}^{\times }$ is −1, it follows that $i^{2}=-1$. Thus i is the quarter-turn element of the shell. □

This proposition clarifies the role of the imaginary unit in the quantum phase factor

$$\exp\left(-i\frac{Et}{\hbar }\right),$$

where *t* is the Object’s relative time in the Subject’s frame. The expression contains two different conversions. The factor *ℏ* converts the conventional energy–time product Et into a phase count. The symbol *i* expresses that phase count in quarter-turn framed-complex notation. The phase count is a frame-readable quantity; i supplies the quarter-turn label in which that count is written. The two roles are distinct: the first is an action-to-phase-count conversion, the second a quarter-turn phase label. The relation between the two Planck constants is the *h*-form pair, one factor from each register: h=2πℏ, with π the stating frame’s own cycle bookkeeping (2π≡−1 in that frame’s ring, whence h≡−ℏ there) and *ℏ* the Carrier’s quantum: *h* is the frame’s per-cycle rebooking of the per-step *ℏ*, derived, not a fifth constant. The residue-level evaluation of the conversion constants on a committed carrier is carried by the quartet analysis of Section 6.

## 4. Modular Unit-Domain Algebra

We now define the finite unit-domain system generated by the two shell charts. The space-like generator is denoted [L], and the time-like generator is denoted [T]. These are formal interface generators attached to the framed shell charts.

**Definition 8**  (Unit-domain assignments)**.** *The symbol [L] denotes the domain generator attached to the additive meridian chart, anchored on the unit *1*. The symbol [T] denotes the domain generator attached to the drive’s phase chart, anchored on the chronon step (one application of x↦gx); the origins 0 and τ carry no units. The two generators record two presentations of one framed shell structure: additive presentation through *1*, and exponential or phase presentation through g. They form the two-generator interface used for dimensional analysis and belong to the physical-interface language rather than to $F_{p}$ itself.*

### 4.1. Finite Scale-Character Exponents

The notation $Z_{p-1}$ denotes the cyclic exponent label system with 4κ=p−1 elements. In the FRC shell it records the finite scale-character exponent attached to the multiplicative phase group $F_{p}^{\times }$: once a primitive frame value $g\in F_{p}^{\times }$ is chosen, the nonzero shell residues are labelled by powers of g modulo the phase period p−1. Thus the time-like domain exponent is a finite scale-character label. The identification itself is constitutive (D): a dimension exponent counts factors of a scale, a character grade indexes a homomorphism of $F_{p}^{\times }$, and this paper identifies the time-like exponent with the latter. The identification is exhibited, not idle: under a drive re-presentation the time exponent transforms as s↦ϵs (Theorem 5(ii)), as a character grade does and as an invariant count of scale factors would not. Once the identification is made, the period p−1 is forced (Remark 3). The space-like exponent is attached instead to the additive meridian period $Z_{p}$, giving the domain-label set $D_{p}$.

**Definition 9**  (Modular unit-domain labels)**.** *Let*

$$D_{p}:=C_{p}\times C_{p-1}.$$

*For $\left(r,s\right)\in D_{p}$, define the modular unit-domain generator $U_{r,s}:={\left[L\right]}^{r}{\left[T\right]}^{s}.$ The neutral domain is $U_{0,0}=1.$*

**Theorem 1**  (Modular unit-domain group)**.** *The set*

$$\left\{U_{r,s}:\left(r,s\right)\in D_{p}\right\}$$

*forms a finite abelian group under the product*

$$U_{r,s}U_{r^{\prime },s^{\prime }}=U_{r+r^{\prime },s+s^{\prime }}.$$

*The inverse of $U_{r,s}$ is $U_{-r,-s}$, and the group is isomorphic to $D_{p}$.*

**Proof.**  Closure follows from componentwise cyclic addition in $D_{p}$: the first component is reduced modulo p, and the second component is reduced modulo p−1. Associativity and commutativity follow from associativity and commutativity of addition in the two cyclic factors. The identity is $U_{0,0}$. The inverse of $U_{r,s}$ is $U_{-r,-s}$, since

$$U_{r,s}U_{-r,-s}=U_{0,0}.$$

The map $\left(r,s\right)\mapsto U_{r,s}$ is a group isomorphism from the additive group $D_{p}$ to the multiplicative domain-label group. □

**Definition 10**  (Homogeneous quantity algebra)**.** *The finite homogeneous quantity algebra generated by the shell domains is*

$$A_{p}:=\underset{\left(r,s\right)\in D_{p}}{⨁}F_{p}U_{r,s}.$$

*The homogeneous fibre of domain (r,s) is*

$$A_{p}^{\left(r,s\right)}:=F_{p}U_{r,s}.$$

*An element of $A_{p}^{\left(r,s\right)}$ has the form*

$$Q=qU_{r,s},\;q\in F_{p}.$$

*The domain label is part of the type of the quantity. Thus $qU_{r,s}$ is not merely the coefficient $q\in F_{p}$; it is the coefficient q read in the homogeneous fibre $F_{p}U_{r,s}$. Addition is defined as physical-interface arithmetic only inside one fixed fibre. A homogeneous fibre $F_{p}U_{r,s}$, together with the scale assignments its frame carries, is an* observer-assigned modular domain*; this is the precise sense of the term throughout the paper.*

**Theorem 2**  (Graded quantity algebra)**.** *The algebra $A_{p}$ is graded by $D_{p}$. More precisely,*

$$A_{p}^{\left(r,s\right)}\;A_{p}^{\left(r^{\prime },s^{\prime }\right)}\subseteq A_{p}^{\left(r+r^{\prime },s+s^{\prime }\right)}.$$

**Proof.**  Let $Q=qU_{r,s}\in A_{p}^{\left(r,s\right)}$ and $R=q^{\prime }U_{r^{\prime },s^{\prime }}\in A_{p}^{\left(r^{\prime },s^{\prime }\right)}$. Define multiplication by

$$\left(qU_{r,s}\right)\left(q^{\prime }U_{r^{\prime },s^{\prime }}\right)=qq^{\prime }U_{r+r^{\prime },s+s^{\prime }}.$$

Since $qq^{\prime }\in F_{p}$, the product lies in $A_{p}^{\left(r+r^{\prime },s+s^{\prime }\right)}$. This proves the grading law. □

The reading is direct: addition is internal to a homogeneous fibre, while multiplication composes domains. The next subsection makes the additive statement exact.

### 4.2. Homogeneous Addition and Neutral-Domain Invariants

The algebra $A_{p}$ is a direct sum of homogeneous domain fibres. This direct-sum structure is the finite FRC version of dimensional homogeneity.

The typed structure prevents cross-domain addition. A value read in the additive order-p domain and a value read in the phase order-(p−1) domain are not summands of one physical-interface quantity. The same obstruction appears after multiplication. If $Q=qU_{r,s}$, then

$$Q^{2}=q^{2}U_{2r,2s}.$$

Thus *Q* and $Q^{2}$ generally lie in different homogeneous fibres, and the formal expression $Q+Q^{2}$ is a direct-sum expression, not a homogeneous physical sum, unless $U_{r,s}=U_{2r,2s}$.

**Definition 11**  (Domain of a homogeneous quantity)**.** *A nonzero element $Q\in A_{p}$ is homogeneous of domain (r,s) if*

$$Q\in F_{p}U_{r,s}\;\mathrm{and}\;Q\neq 0,$$

*in which case we write ${\mathrm{dom}}_{p}\left(Q\right)=\left(r,s\right)$. The zero element is assigned a domain only by context, since it belongs to every homogeneous fibre.*

**Theorem 3**  (Fibrewise addition)**.** *Let*

$$Q_{j}=q_{j}U_{r_{j},s_{j}}\;\left(1\leq j\leq N\right)$$

*be homogeneous elements of $A_{p}$. The sum $Q_{1}+\cdots +Q_{N}$ is either zero or homogeneous if all nonzero summands have the same domain. In general, the component of the sum in the fibre $F_{p}U_{a,b}$ is the sum of all summands whose domain is (a,b). Hence the sum is nonzero and homogeneous of domain (r,s) exactly when its (r,s)-component is nonzero and all other fibre components vanish. As a physical-interface addition rule, a sum is admissible without cross-domain cancellation only when all nonzero summands are supplied in the same domain.*

**Proof.**  If all nonzero summands lie in the same fibre $F_{p}U_{r,s}$, then their sum also lies in that fibre; if the sum is nonzero, it is a homogeneous element of domain (r,s).

For the general statement, group the summands by their domain labels. Since $A_{p}$ is a direct sum, every element has a unique decomposition into components in the fibres $F_{p}U_{a,b}$. The component in fibre (a,b) is precisely the sum of the terms $q_{j}U_{a,b}$ with $\left(r_{j},s_{j}\right)=\left(a,b\right)$. Therefore the total sum is homogeneous of domain (r,s) exactly when the (r,s)-component is nonzero and every component in a different fibre is zero. The final admissibility statement is the corresponding physical-interface rule that addition is defined inside one homogeneous fibre. □

Thus, a formal expression such as

$$3{\left[L\right]}^{2}+5{\left[L\right]}^{3}$$

may be written in the direct sum, but it is not one homogeneous physical quantity. The usual rule that areas cannot be added to volumes is the fibrewise addition theorem in this setting.

**Theorem 4**  (Neutral-domain invariant criterion)**.** *Let $Q_{1},\ldots ,Q_{N}$ be nonzero homogeneous quantities with ${\mathrm{dom}}_{p}\left(Q_{j}\right)=\left(r_{j},s_{j}\right)$. For exponent choices $k_{j}$, define the monomial*

$$M=\prod_{j=1}^{N}Q_{j}^{k_{j}}.$$

*Then*

$${\mathrm{dom}}_{p}\left(M\right)=\sum_{j=1}^{N}k_{j}\left(r_{j},s_{j}\right).$$

*In particular, M is a neutral-domain invariant if and only if*

$$\sum_{j=1}^{N}k_{j}\left(r_{j},s_{j}\right)=\left(0,0\right)$$

*in $D_{p}$.*

**Proof.**  Write $Q_{j}=q_{j}U_{r_{j},s_{j}}$. By repeated use of the multiplication rule,

$$Q_{j}^{k_{j}}=q_{j}^{k_{j}}U_{k_{j}r_{j},k_{j}s_{j}},$$

and multiplying these terms gives

$$M=\left(\prod_{j=1}^{N}q_{j}^{k_{j}}\right)U_{\sum_{j}k_{j}r_{j},\sum_{j}k_{j}s_{j}}.$$

The domain formula follows, and the neutral-domain condition is exactly the statement that the resulting domain label is (0,0). □

This theorem is the modular FRC counterpart of the usual dimensionless-invariant condition in classical dimensional analysis. The analogy with Noether’s theorem is structural rather than variational: the present paper has no action principle, but it uses the same guiding logic that admissible transformations determine invariant physical content [5].

### 4.3. Admissible Reframings and Covariance

The transformations under which the domain system must be covariant are the frame changes of the shell itself, and they are fixed here once; every later invocation of “canonical” or “frame-covariant” refers to this definition.

**Definition 12**  (Admissible reframing; the two actions)**.** *An admissible reframing of the framed shell $F_{p}\left(\tau ;0,1,g\right)$ is a triple (a,m,ϵ): a translation of the spatial origin, x↦x+a with $a\in F_{p}$; a dilation of the unit, 1↦m with $m\in F_{p}^{\times }$; and a re-presentation of the drive, $g\mapsto g^{\epsilon }$ with $\epsilon \in Z_{p-1}^{\times }$. A reframing acts twice over, and the two actions are kept distinct throughout.* The passive (coefficient) action *is defined on the integer-lifted labels $\left(r,s;j\right)\in Z^{3}$ of Definition 14, where integer exponents make it well defined: translations act trivially, and the dilation multiplies the coefficient of a lifted-label-(r,s;j) quantity by the character $m^{-r}$, multiplicative in r over Z. The finite label (rmodp,smodp−1) is the modular projection of the lift; the passive character is a function of the lift, not of the projection, and only its bounded-window restriction descends (the declared $Z_{p}$ refinement carries no character). The active (pushforward) action carries the drive re-presentation: a scale character of grade s counted against the step of g is the character of grade ϵs against the step of $g^{\epsilon }$, and a re-presentation that reverses the quarter-orientation flips the sign convention of the crossing degree exactly as it sends i to −i. The action on the lift is therefore*

$$\left(r,\;s;\;j\right)\;⟼\;\left(r,\;\epsilon s;\;\sigma \left(\epsilon \right)\;j\right),\;\sigma \left(\epsilon \right)=\left\{\begin{array}{cc} +1, & \epsilon \equiv 1\;(\mathrm{mod}\;4),\\ -1, & \epsilon \equiv 3\;(\mathrm{mod}\;4), \end{array}\right.$$

*with no coefficient factor; equivalently, crossing degrees are counted against the frame’s* own *oriented flag generator, which the re-presentation carries to $i_{D}^{\pm 1}$. Formally, the active action is the group action of $Z_{p-1}^{\times }$ on the realized coordinate $u=s+j\kappa \in C_{p-1}$, u↦ϵu, where residue classes act and composition is automatic; the displayed integer formula is a* lift representative *of it, exact as the congruence ϵs+σ(ϵ)jκ≡ϵ(s+jκ)(mod4κ), since 4∣(ϵ−σ(ϵ)): representatives compose only up to the modular reduction, which is where the action lives. The sign σ is multiplicative modulo four, and the realized action is linear, so the homomorphism property of Definition 14 survives.*

**Theorem 5**  (Window covariance)**.** *Fix a bounded window |r|,|s|≤H<2κ (below the half-period, the same horizon as Theorem 10’s 4κ>2H) and restrict to the flag-free sector j=0. The restriction is necessary, for a sharper reason than covariance: on the realized lattice, invariance under every re-presentation holds exactly on the cardinal pair, u=s+jκ∈{0,π} (the argument of* (ii) *applied to the realized coordinate), so a flagged label is invariant precisely when its realization lands on that pair—(0,κ;1) realizes π and (0,−κ;1) realizes *0*, invariant without being neutral, the flag content absorbed by realization. On the pure flag s=0 the odd sectors are never invariant (ϵ=−1 carries the realized label of (0,0;1) from κ to 3κ) and j≡2 is the invariant half-period ${\left[T\right]}^{\pi }$, the cardinal −1. Outside j=0, invariance therefore no longer certifies neutrality, which is exactly what* (iii) *requires. Then:* (i) *on the lift the passive character is multiplicative and fibrewise constant with no window hypothesis, so it commutes with the product of Theorem 2 and the fibrewise addition of Theorem 3; the window enters only in the converse: an in-window monomial has trivial dilation character for every $m\in F_{p}^{\times }$ exactly when r=0, since m primitive forces (p−1)∣r and |r|≤H<2κ<p−1 forces r=0.* (ii) *The pushforward action preserves the fibre structure and fixes an in-window time label for every admissible ϵ exactly when s=0; on the full realized lattice its invariant sublattice in the time-exponent factor is {0,π} (the neutral label and the half-period, the cardinal pair {1,−1} shared by every frame), while the quarter-turn labels ±κ are fixed or exchanged as ϵmod4∈{1,3}, exactly as the residue quarter-turn ±i is.* (iii) *Consequently an in-window homogeneous quantity of the flag-free sector reads identically in every admissible frame exactly when its label is neutral: window invariance is window neutrality, on exactly the count-valued sector (ledger:C5).*

**Proof.**  (i) On the lift, $m^{-r}m^{-r^{\prime }}=m^{-(r+r^{\prime })}$ with integer exponents, so the character is multiplicative and fibrewise constant. If $m^{-r}=1$ for a primitive *m*, the order p−1 of *m* divides *r*; with |r|≤H<p−1 this gives r=0. (ii) The pushforward is s↦ϵs on $C_{p-1}$. If ϵs≡s for all $\epsilon \in Z_{p-1}^{\times }$, then taking ϵ=−1 (always admissible, since gcd(4κ−1,4κ)=1) gives 2s≡0(mod4κ), so s∈{0,π} in $C_{p-1}$; conversely π=2κ is invariant because every admissible ϵ is odd. The same argument applied to the realized coordinate u=s+jκ classifies invariance on the flagged sectors: ϵu≡u for every admissible ϵ exactly when u∈{0,π}. For an in-window label, s=0. The flag subgroup {0,κ,2κ,3κ} is preserved as a set since ϵκ≡(ϵmod4)κ(mod4κ) with ϵ odd, giving ϵκ∈{κ,3κ}. (iii) A quantity fixed by every reframing is fixed in particular by all dilations and all re-presentations; by (i) and (ii) its window label is (0,0), and conversely the neutral label has trivial character and fixed realized label. □

**Remark 3**  (What the action forces, and what is declared)**.** *The pushforward action of $Z_{p-1}^{\times }$ is an action on $C_{p-1}$: the time-exponent label system is the phase period p−1, forced by the action the drive re-presentations actually have. The dilation character, defined on the lift, determines the space exponent only modulo p−1 (the order of the dilation group); the refinement of the [L]-period to p, the label lattice $D_{p}=C_{p}\times C_{p-1}$, is a declared identification (D): the space exponent is labelled by the additive meridian’s native coordinate period, the cyclic depth $Z_{p}$ of the chart the generator is anchored on. The bicyclic lattice thus rests on two declared identifications of different downstream status: the character-grade identification of the time exponent (D, exhibited by the covariance of Theorem 5(ii)), from which the period p−1 is then forced, and the $Z_{p}$ refinement of the space period, which no forcing follows; it is used in what follows under that status. The window bound H<2κ is sharp: at H=π the label ${\left[T\right]}^{\pi }$ enters the window, invariant and non-neutral: below the half-period, invariance is neutrality; at the half-period, the one extra invariant is the cardinal −1. The invariant pair {1,−1} of Theorem 5(ii) is the domain face of a shell fact: the cardinal pair is frame-absolute while the quarter-turns are orientation data: the covariance of the flag mirrors the covariance of i because both are the same order-four structure read on two faces.*

**Remark 4**  (Two temporal transformations, and the atomic chronon)**.** *Classical temporal unit change (seconds to milliseconds) keeps the label ${\left[T\right]}^{s}$ fixed and rescales the coefficient. In the shell that transformation is nominal scale re-assignment within a fixed drive presentation: it changes the reading, not the shell (Section 2), it is label-preserving by construction, and classical exponent covariance holds under exactly that identification. The drive re-presentation $g\mapsto g^{\epsilon }$ is a different object, a strictly finite transformation with no free-abelian counterpart, and exponent invariance under it fails by design and is not claimed. The absent temporal coefficient character is not an omission but a theorem: the chronon is atomic, the finite drive admits no scalar sub-division of its step, and the would-be character $\epsilon^{-s}$ fails composition outright (the verification suite retains the counterexample as a regression). The classical $\epsilon^{s}$ covariance of temporal unit change is recovered in the window reading, where scale re-assignment is nominal and the drive presentation is held fixed.*

**Remark 5**  (The exponent lattice is not the spacetime)**.** *The two periods are the orders of the two cyclic actions a prime field carries: every cyclic subgroup of the frame group x↦ax+b is, up to reframing, a subgroup of the translations (a group of order p) or of the dilations (of order p−1), and there is no third, since an affine map with a≠1 fixes b/(1−a) and is conjugate to x↦ax by the translation carrying that point to 0; the framework’s realisation reads the drive as the observer’s time and the additive meridian as its space [24,38]. The coordinate charts carry the periods first (Definition 4, Proposition 1); the lattice $D_{p}$ takes the time period from the drive’s action, forced once the character-grade identification is made, and the space period from the meridian’s coordinate period, declared (Remark 3). It is the exponent lattice of the dimensions, not the spacetime: as in the classical M-L-T treatment, [L] and [T] are independent generators and no Lorentz transformation acts on the dimension group. A boost mixes coordinates within one fibre, x with ct, with c the conversion, and touches no label; boosts are not among the reframings of Definition 12, under which the labels are covariant on the window (Theorem 5); the Lorentzian structure of the framework is [38]. The periods differ only at the wrap, which no window reaches; the window reads as the Z-lift of Corollary 9, as classically. The order-four subgroup exists by 4∣p−1 alone, which holds on every shell by p=4κ+1, the condition p≡1(mod4) that gives the shell its quarter-turn i; what the odd meridian period adds is that the space factor carries no element of even order, so the lattice has a single element of order two, no Klein four-group, and the order-four subgroup is unique and lies in the time factor (Theorem 7, Corollary 1): the asymmetry is structural at the level of the charts and rests on the declared refinement at the level of the exponents.*

## 5. The Four Domains and the Unit Flag

Energy is the first derived domain that differs substantially from the standard mechanical presentation. In the shell-domain reading, energy is not first introduced as mass times velocity squared: its domain is fixed by the Planck relation together with the shell’s four-domain Fourier structure. A *finite-domain reading* of a constant is the pair of its domain label and its representation in the shell’s cycle-order data; the results of this section and Section 7, summarized in Table 1, instantiate this definition for *h*, *ℏ*, *c*, and *G*.

**Definition 13**  (Temporal recurrence domain)**.** *A frequency f counts phase cycles per chronon step. Its domain is*

$$\left[f\right]={\left[T\right]}^{-1}.$$

*Verified in ledger:D1.*

The physical realisation of energy is the Planck relation E=hf. This relation does not merely compare two measured quantities: it says that every energy value is a temporal recurrence rate, read through one universal constant *h*. In the modular unit-domain algebra, domains distinguish comparison fibres, and a single universal conversion constant cannot introduce a new free domain generator: any such generator would be carried entirely by *h*, but *h* is the same scale for every frame-local expression. The domain of *h* therefore lies not in a new generator but in the torsion the bicyclic lattice already carries, fixed by the shell’s Fourier structure, established below.

A complete phase turn can be counted in two equivalent ways: in cycle units it is one cycle; in angular units it is the real 2π of the chart. In a finite cyclic phase representation the complete turn is represented by the order of the phase cycle, and for the shell phase cycle that order is 4κ=2π=p−1. Here π is the shell’s half-period 2κ, the residue with $g^{\pi }=-1$ (Definition 2), and the chain is that residue’s definition, not an identity with the real number: when a phase exponent *k* is read as the fraction k/(p−1) of a full turn on the circle, the chart of the phase cycle, the residue identity $g^{\pi }=-1$ reads as exp(iπ)=−1, the half-period at the half-turn, and the reading is exact at every finite p. Propagation over a finite cycle of order *N* is written

$$\exp\left(-i2\pi \frac{nk}{N}\right).$$

Setting N=p−1 and comparing with

$$\exp\left(-i2\pi \frac{Et}{h}\right)$$

shows that, when *E* and *t* are phase labels in $Z_{p-1}$, the divisor slot that *h* occupies is filled by the full phase-cycle order: the complete-cycle count carried by *h* is p−1≡−1(modp), the same count that represents 2π. Thus *h* converts frequency measured relative to the chosen time scale into frequency measured relative to the complete phase cycle: its counting face. The count fixes the magnitude of the conversion, not the constant’s residue; the residue face is carried by the quartet analysis of Section 6. Using angular frequency gives ω=2πf and E=ℏω: because *h* and 2π are represented by the same complete-cycle count, the two Planck constants differ by the half-turn, 2π≡−1, and carry one and the same modular domain. The relation h=2πℏ is read on each face with that face’s 2π, the real number at the unit face (Corollary 3) and the doubled half-period on the shell (Remark 14): one relation, not one identity between a residue and a real number.

**Theorem 6**  (Chart duality of the domain lattice)**.** *Define the chart duality on domain labels by*

$$\delta_{S}:\;D\;⟼\;D^{-1}.$$

*Then $\delta_{S}$ is an involution of the modular unit-domain group, and its orbit on the two generators is the four-domain structure of Figure 2:*

$$\delta_{S}\left[L\right]={\left[L\right]}^{-1}\;(\mathrm{the}\;\mathrm{momentum}\;\mathrm{chart}),\;\delta_{S}\left[T\right]={\left[T\right]}^{-1}\;(\mathrm{the}\;\mathrm{energy}\;\mathrm{chart}).$$

*Verified in ledger:D1.*

**Proof.**  $\delta_{S}^{2}=\mathrm{id}$ is immediate from the group law of Theorem 1, and the $\delta_{S}$-images of the two generators are their inversions. The four labels $\left[L\right],{\left[L\right]}^{-1},\left[T\right],{\left[T\right]}^{-1}$ are pairwise distinct in $D_{p}$ whenever κ≥1, so the orbit has exactly the four members shown in the figure. □

Each dual pair carries two tiers of reading, and the tiers are the registers of Section 2; Figure 2 renders the four domains in both, one row per register. The top row is the Subject register: the observer’s own coordinates on the dual charts are the wavenumber and the frequency, pure counts against the observer’s chosen units, carrying the flag-free labels ${\left[L\right]}^{-1}$ and ${\left[T\right]}^{-1}$. The bottom row is the Carrier register: the same domains with the dual charts named by their unitful readings, momentum and energy, anchored at the substrate, whose absolute value no bounded observer expression closes, carrying the quartet of horizons (Section 6). The marker that separates the two rows, the register crossing, is the substance of the Planck constant, and it lives in the torsion the domain lattice itself carries. In map form: the top row is the chart duality $\delta_{S}:D\mapsto D^{-1}$ of Theorem 6, flag-free count duality within the Subject register; the bottom row’s primal-to-dual passage is the *crossed duality* $\delta_{C}:=i_{D}\;\delta_{S}$, defined once the flag is established below: an involution, since $\delta_{C}^{2}\left(D\right)=i_{D}{\left(i_{D}D^{-1}\right)}^{-1}=D$.

**Theorem 7**  (The internal flag)**.** *The domain lattice $D_{p}=C_{p}\times C_{p-1}$ contains exactly one subgroup of order four,*

$$Q_{4}^{D}=\langle {\left[T\right]}^{\kappa }\rangle =\left\{1,{\left[T\right]}^{\kappa },{\left[T\right]}^{2\kappa },{\left[T\right]}^{3\kappa }\right\},$$

*and it lies entirely in the time-exponent factor. Define the* unit flag

$$i_{D}:={\left[T\right]}^{\kappa },\;i_{D}^{4}=1,\;i_{D}^{2}={\left[T\right]}^{\pi },$$

*with the orientation (the choice of ${\left[T\right]}^{\kappa }$ over ${\left[T\right]}^{-\kappa }={\left[T\right]}^{3\kappa }$) following the chart convention of the residue quarter-turn $i=g^{-\kappa }$. The flag is the capacity element of the shell’s own exponent lattice, the fractional Fourier cycle $F^{4}=\mathrm{id}$ read on domains, and its square is the half-period element π=2κ (ledger:D2).*

**Proof.**  An element $\left(r,s\right)\in C_{p}\times C_{p-1}$ has order four exactly when 4(r,s)=(0,0) and 2(r,s)≠(0,0). Since p is an odd prime, 4r≡0(modp) forces r=0: the meridian factor is torsion-free below its full period and contributes nothing. In the cyclic factor $C_{p-1}=C_{4\kappa }$ the elements of order four are the two generators κ and 3κ of the unique order-four subgroup of a cyclic group whose order is divisible by four. Hence $D_{p}$ has exactly one order-four subgroup, $\langle {\left[T\right]}^{\kappa }\rangle$, with two possible generators (a subgroup of order four not generated by an element of order four would be a Klein four-group, and $D_{p}$ has a single element of order two, (0,2κ), since p is odd); the stated orientation convention selects ${\left[T\right]}^{\kappa }$. Covariance: under the pushforward action of Definition 12 the capacity exponent maps to ϵκ≡κ·(ϵmod4)(mod4κ), so the subgroup is preserved and the generator is fixed or conjugated to its inverse exactly as i is: one orientation convention serves the residue and domain faces alike. □

**Remark 6**  (The transform behind the cycle)**.** *The Fourier structure invoked here is the shell’s own [39]: on the module $F_{p}^{\Phi }$ of vectors over the phase cycle $\Phi =C_{p-1}$, with coefficients in $F_{p}$, the shell Fourier matrix W has entries $W_{jk}=g^{jk}$. (The source construction names this index cycle the meridian cycle; in the present paper’s taxonomy the meridian is the additive order-p factor, so the phase name is used throughout.) The four-cycle is a geometric sum: ${\left(W^{2}\right)}_{jl}=\sum_{k\in \Phi }g^{k(j+l)}$ equals p−1≡−1 when j+l≡0(modp−1) and *0* otherwise, so $W^{2}=-J$ with J the index inversion (parity); the frame’s quarter-turn normalises it, F:=iW, whence $F^{2}=i^{2}W^{2}=J$ and $F^{4}=J^{2}=\mathrm{id}$, with $F^{2}=J\neq \mathrm{id}$. The fractional family $F^{\left[s\right]}$, additive in the phase index and faithful on the full cycle, is [39]. No additive p-th root of unity is invoked anywhere, and no transform on the additive chart is constructed or needed: the operator is phase-cycle data, internal to the shell, implementing the time–frequency conjugate pair; the space–momentum pair is defined by the label duality of Theorem 6 and anchored by the same single crossing quantum (Definition 15: one crossing, one action). The four-domain structure is the cardinal skeleton $\left\{F^{\left[0\right]},F^{\left[\kappa \right]},F^{\left[2\kappa \right]},F^{\left[3\kappa \right]}\right\}=\left\{\mathrm{id},F,J,F^{3}\right\}$ read on domains.*

**Proposition 3**  (The lift: the flag records the quarter the chart duality forgets)**.** *Read the operator cycle of Remark 6 on charts and on domain labels. On charts the cardinal skeleton acts through the chart duality: $F^{\left[\kappa \right]}=F$ carries each conjugate chart to its dual, and $F^{\left[2\kappa \right]}=J$ fixes each chart setwise, so the chart action of the skeleton factors through the quotient $Z_{4}\to Z_{2}$. On the domain lattice the record map $\rho :F^{\left[u\right]}\mapsto {\left[T\right]}^{u}$(u∈{0,κ,2κ,3κ}) is index-preserving—the flag exponent κ is the fractional index of one quarter—hence canonical rather than chosen, and it is an isomorphism onto the flag subgroup $\left\{1,i_{D},i_{D}^{2},i_{D}^{3}\right\}$ of Theorem 7; the chart action is the record read modulo the parity element $i_{D}^{2}={\left[T\right]}^{\pi }$. The operator has order four, its chart shadow order two, and the flag is exactly the datum that records the forgotten factor: one register crossing is one quarter because a crossing steps along the operator cycle, of which the chart duality is only the shadow. The Carrier face states the same order independently: the crossing quantum’s pin $\hbar^{2}\equiv -1$ (Theorem 9) makes two crossings the half-turn and four the identity, never two. The premise of Proposition 5(i) is thereby argued rather than declared, and ${\left[T\right]}^{\pi }$, of order two, cannot carry the crossing: its cycle closes where the operator’s and the pin’s visibly do not (ledger:D3).*

**Proof.**  On charts, *F* exchanges the two conjugate phase charts (the time–frequency pair it implements) and *J* inverts coordinates within each chart, exchanging none, so the skeleton’s chart action is smod2; the spatial pair reads the same action through the label duality and the one-crossing anchoring of Definition 15. The record map is index-preserving and sends generator to generator, hence an isomorphism of cyclic groups of order four; composing it with the quotient by $\left\{1,i_{D}^{2}\right\}$ returns smod2, the chart action, so the diagram commutes and the kernel of the forgetting is exactly {id,J}. The four-cycle is the geometric-sum computation of Remark 6; the fractional family is [39]; the uniqueness of the order-four subgroup is Theorem 7; the pin statement is Theorem 9. The python check of ledger:D3 verifies the operator four-cycle, the order-two chart shadow, and the pin orders in exact arithmetic on (13,233) and on the laboratory Carrier. □

**Corollary 1**  (No flag of space)**.** *The meridian factor $Z_{p}$ contains no element of order four, so no spatial monomial ${\left[L\right]}^{r}$ and no mixed element with r≠0 is itself of order four: every order-four element of $D_{p}$ lies in the time-exponent factor. (Powers of mixed elements can land on the flag, Proposition 4, but no such element is of order four.) The register crossing can only be carried on the time-exponent lattice; the asymmetry between the charts is structural, not conventional (ledger:D2).*

**Proposition 4**  (Meridian transport onto the flag)**.** *The two periods interfere: ${\left(\left[L\right]\;{\left[T\right]}^{\kappa }\right)}^{p}=i_{D}$ (ledger:D2).*

**Proof.**  The meridian component wraps, p≡0(modp), while the phase component advances by pκ=(4κ+1)κ≡κ(mod4κ). The identity uses both periods at once (the meridian wrap and the phase period) and is, with the confinement of the flag to the time-exponent factor (Theorem 7, Corollary 1, whose arguments use the odd meridian period), a visible act of the declared $Z_{p}$ refinement in this paper: the meridian period transports mixed powers onto the flag. □

**Definition 14**  (Crossing degree)**.** *Dimension bookkeeping tracks, alongside the free exponents, an integer* crossing degree *j∈Z: the signed number of register crossings in a quantity’s formation. The full label of a quantity is (r,s;j), realized on the shell as the lattice element ${\left[L\right]}^{r}{\left[T\right]}^{s}i_{D}^{\;j}=U_{r,\;s+j\kappa }$, with the* sector *jmod4: the residue class of the crossing degree modulo four, the position on the flag subgroup. The realization map is a homomorphism, $\left(r,s;j\right)+\left(r^{\prime },s^{\prime };j^{\prime }\right)\mapsto U_{r+r^{\prime },\;s+s^{\prime }+\left(j+j^{\prime }\right)\kappa }$, so the sector of a product is the sum of sectors: the flag grading, with additivity by construction.*

**Proposition 5**  (The unit flag)**.** *The Planck constants carry the flag:*

$$\left[h\right]=\left[\hbar \right]=i_{D}.$$

**Proof.**  *Realisation content* (R), stated and constitutive: the relations E=hf and p=ℏk each relate two readings of one quantity—the observer’s count (*f*, *k*) and the unitful measure (*E*, *p*), whose absolute unit is fixed by the substrate. Each is a unit-face relation between observer-chart magnitudes; its exact content in the domain algebra is the label equality, and no coefficient arithmetic crosses registers (Section 2 excludes cross-register formulas; Remark 13 states what registration permits). The conversion domain is (i) of order four, one quarter of the Fourier cycle per crossing, closing on the fourth: the shell’s fractional Fourier cycle $F^{4}=\mathrm{id}$ [25,39], the order-four premise argued by the lift of Proposition 3; (ii) horizon-inaccessible, a register crossing rather than a bounded rescaling within the observer’s window (Corollary 2); (iii) canonical and covariant under the pushforward action of Definition 12. The realisation stands with its explicit falsifier: one empirically closed expression that adds an observer count to a unitful measure without the conversion quantum (an energy to a frequency, a momentum to a wavenumber) would kill it. *Proof over the realisation*: by Theorem 7 the lattice contains exactly one order-four subgroup, it is covariant, and its generator is fixed by the orientation of i: the flag $i_{D}={\left[T\right]}^{\kappa }$ is the unique admissible conversion domain, and nothing beyond Theorem 7 is used. Since h=2πℏ with 2π a pure cycle count, [h]=[ℏ]. □

**Corollary 2**  (The absolute unit is horizon-inaccessible)**.** *The flag sits at the capacity: its [T]-exponent is κ, the quarter-period. Classical dimensional analysis operates inside a bounded exponent horizon H far below the capacity (Theorem 10 with H≪κ); within any such window no monomial expresses the flag, and a free-abelian dimension group generated by in-window symbols contains no order-four element at all. A torsion-free dimensional framework therefore cannot register the flag structurally; it discards it and adjoins a free primitive in its place: the third generator M of classical M-L-T bookkeeping is this torsion-free surrogate, as the closure $\left[M\right]=i_{D}\;{\left[L\right]}^{-2}\left[T\right]$ exhibits. The absolute value of the unit, the residue face of the flag, is fixed by the totality: Ω-hard (Section 6) (ledger:D6).*

**Proof.**  Within a window |r|,|s|≤H<κ the realized flag exponent κ is out of reach, so no in-window monomial equals $i_{D}$; and the free abelian group on in-window generators is torsion-free, so it contains no image of the order-four subgroup except through a quotient that discards it. A framework that discards the flag collapses the unitful measures onto the observer counts; the alternative is a new free generator, and classical dimensional analysis takes that route: the mass proposition of Section 7 identifies the replacement, $\left[M\right]=i_{D}\;{\left[L\right]}^{-2}\left[T\right]$. □

**Remark 7**  (Near-saturation visibility)**.** *The inaccessibility is horizon-relative, and deliberately so: an observer whose exponent window approaches the capacity, κ≤H<2κ, would find the flag expressible as the chart monomial ${\left[T\right]}^{\kappa }$: at saturation the register crossing becomes visible as a time-exponent of the observer’s own chart. Classical bookkeeping is torsion-free precisely because it reads the lattice far below the wrap, where the lattice looks free; the flag is what that reading truncates (ledger:D6).*

**Proposition 6**  (Energy and momentum as flagged readings)**.** *The unitful readings carry the flag over the observer’s counts:*

$$\left[E\right]=\left[h\right]\left[f\right]=i_{D}\;{\left[T\right]}^{-1},\;\left[p\right]=\left[\hbar \right]\left[k\right]=i_{D}\;{\left[L\right]}^{-1},$$

*one quantity per pair, the two labels differing exactly by the flag. On the realized lattice the flagged readings sit one capacity step above their counts, $\left[E\right]={\left[T\right]}^{\kappa -1}$ and $\left[p\right]={\left[L\right]}^{-1}{\left[T\right]}^{\kappa }$, with crossing degree one. The flagged readings are mutually comparable by chart conversions, [E]=[p][c], and discarding the flag sends each to its observer count, [E]↦[f], [p]↦[k].*

**Proof.**  Direct from E=hf, p=ℏk, and Proposition 5; $\left[p\right]\left[c\right]=i_{D}{\left[L\right]}^{-1}\left[L\right]{\left[T\right]}^{-1}=i_{D}{\left[T\right]}^{-1}=\left[E\right]$. The projection statement is immediate. □

**Proposition 7**  (Action and phase counts)**.** *The energy–time product carries the flag itself,*

$$\left[S\right]=\left[E\right]\left[T\right]=i_{D}=\left[h\right],$$

*and every phase count is exactly neutral:*

$$\left[E\right]\left[T\right]\;{\left[\hbar \right]}^{-1}=1,\;\left[p\right]\left[L\right]\;{\left[\hbar \right]}^{-1}=1.$$

**Proof.**  $\left[E\right]\left[T\right]=i_{D}{\left[T\right]}^{-1}\left[T\right]=i_{D}$, and $\left[p\right]\left[L\right]=i_{D}$; dividing by $\left[\hbar \right]=i_{D}$ is exact neutrality in both cases. □

**Remark 8**  (Units as reciprocal relations)**.** *A unit of measure is constitutively a relation between two count systems: a measure expresses counts of one frame per count of another, and no single count system carries a unit of its own. The unit flag is the domain form of this reciprocity, the register crossing of Section 2 made algebraic. Its residue face is the quarter-turn, the shell’s crossing between a chart and its dual; its domain face is the capacity element ${\left[T\right]}^{\kappa }$; its counting face is the complete cycle, p−1 observer steps per one full turn of the substrate; and the crossing degree of any derived quantity counts the net substrate-legs in its defining ratio: zero for intra-observer ratios such as speed, frequency, and wavenumber, one for the unitful measures, minus one for the coupling G. That the flag has no expression in the observer’s dimensions (Corollary 2) is why units present to the bounded observer as primitive conventions rather than derived quantities, the classical mass primitive standing in for the flag.*

Neutrality of the phase counts, not of the constants, is the invariant content: the conversion the Planck constants effect carries the lattice quarter-turn. In conventional units, *h* fixes the energy scale assigned to one complete phase cycle, while *ℏ* fixes the corresponding angular phase scale; neither is the finite quarter-turn label i. The roles are different, and the phase factor has two components:

h,ℏ:conventionalenergy-actionunits⟶phasecount,

and

i:phasecount⟶framed-complexquarter-turnlabel.

This separation explains why energy can be meaningful without spatial propagation: a frame-localized shell-vector pattern may carry energy through an internal temporal recurrence rate in a chosen frame. In classical relativity the same energy domain can be read through spacetime motion, stress-energy, or gravitational coupling; the domain is the same, the frame-readable carrier expression differs.

**Remark 9**  (Classical energy readings)**.** *The Planck relation is the unique interface at which the energy domain can be introduced without a mass primitive. The shell supplies exactly two native scale assignments, the unit and the chronon step, hence exactly two domain generators; a classical definition of energy through $E=\frac{1}{2}mv^{2}$ would presuppose an independent mass domain, which the two-generator system does not contain. Once $\left[m\right]=\left[E\right]{\left[v\right]}^{-2}$ defines the mass domain (Section 7), every classical energy expression becomes a consistency check on the same fibre: the kinetic-energy identity closes as $\left[m\right]{\left[v\right]}^{2}=i_{D}{\left[T\right]}^{-1}=\left[E\right]$ (Example 2), and the relativistic reading $E=mc^{2}$ closes identically. On either route the constants are the same scale conversions.*

The phase-count language also touches the informational tradition in quantum foundations: Wheeler ties physical facts to finite distinctions, and Landauer stresses that information requires physical embodiment [17,40]. Here the use is narrower: phase count is a finite physical-interface quantity, not a claim that the full FRC shell is a digital computation.

## 6. The Carrier Quartet: Fundamental Units as Horizons

The flag of Section 5 marks the crossing from the observer’s counts to measures anchored at the substrate. This section identifies the anchors. Each of the four representation domains carries a Carrier-scale horizon, and the four horizons are the fundamental physical units; the four physical constants follow from them uniquely.

The section is the holographic reading of Remark 1 at the unit face. On a finite holographic substrate every substrate-anchored measure is boundary data: what the observer meets at the edge of its own window, never inside it. The word horizon is therefore literal. The four Planck units are the four readings of the one capacity at the observer’s horizon, one per representation domain, and the quartet theorem below states that these boundary data determine the conversion constants completely: two anchored horizons and one crossing quantum, nothing else.

Two determinations run through the section, and they are independent. The unit face is determined by the quartet: two anchored horizons and one crossing quantum fix the constants’ magnitudes, domains, and flag positions (Definition 15 through Corollary 4). The residue face is determined by the Carrier alone: on every admissible Ω the defining congruences of Theorem 9 have their canonical root pairs, with no input from the quartet, and $G=-c^{2}=\left(\Omega -1\right)/2$ holds in every prime field where the roots exist. Theorem 9 is that Ω-uniform assignment, and its Ω-blindness is a feature: the naming of the constants does not depend on which admissible Carrier is instantiated. What joins the two faces is the realisation of Definition 15 and the residue assignments below, tagged as such.

**Definition 15**  (The quartet)**.** *The primitive data are three: the two* anchored horizons *, $\ell_{P}$ (space) and $t_{P}$ (time), the substrate-fixed extreme marks of the observer’s two primal charts, flag-free re-anchorings of the charts themselves, $\left[\ell_{P}\right]=\left[L\right]$, $\left[t_{P}\right]=\left[T\right]$; and the* crossing quantum *ℏ, the substrate-anchored value of one register crossing, $\left[\hbar \right]=i_{D}$. The dual horizons are their crossed images,*

$$p_{P}:=\frac{\hbar }{\ell_{P}}\;\left(\mathrm{momentum}\right),\;E_{P}:=\frac{\hbar }{t_{P}}\;\left(\mathrm{energy}\right).$$

*(R: the anchoring of the two primal horizons and of the crossing is the realisation; everything below is a theorem over it.)*

**Remark 10**  (Domain completeness)**.** *The four horizons occupy the four representation domains of Figure 2 (bottom row, the Carrier register), one per domain: $\left[p_{P}\right]=i_{D}\;{\left[L\right]}^{-1}$ and $\left[E_{P}\right]=i_{D}\;{\left[T\right]}^{-1}$ by construction, so the quartet $\left\{\ell_{P},t_{P},p_{P},E_{P}\right\}$ is domain-complete, with flag positions (0,0,1,1). The completeness is a direct reading of Definition 15, recorded here rather than claimed as a theorem.*

The passage from the Subject row to the Carrier row of Figure 2 is therefore not uniform across the square, by construction: the primal horizons re-anchor the observer’s own charts without crossing, while the dual horizons sit one flag above their counts. On the dual charts the two rows differ by exactly what the Planck constant does; on the primal charts, by anchor alone.

**Remark 11**  (One crossing, one action)**.** *The quartet satisfies the single internal identity $\ell_{P}\;p_{P}=t_{P}\;E_{P}\;\left(=\hbar \right)$, immediately from Definition 15: $\ell_{P}p_{P}=\ell_{P}\cdot \hbar /\ell_{P}=\hbar =t_{P}\cdot \hbar /t_{P}=t_{P}E_{P}$. The content the identity carries is located in the definition itself: there is one crossing quantum, not one per conjugate pair, because the flag subgroup has one generator (Theorem 7) and ℏ is anchored once, on the crossing, not on a chart (ledger:E2).*

**Figure 4:**
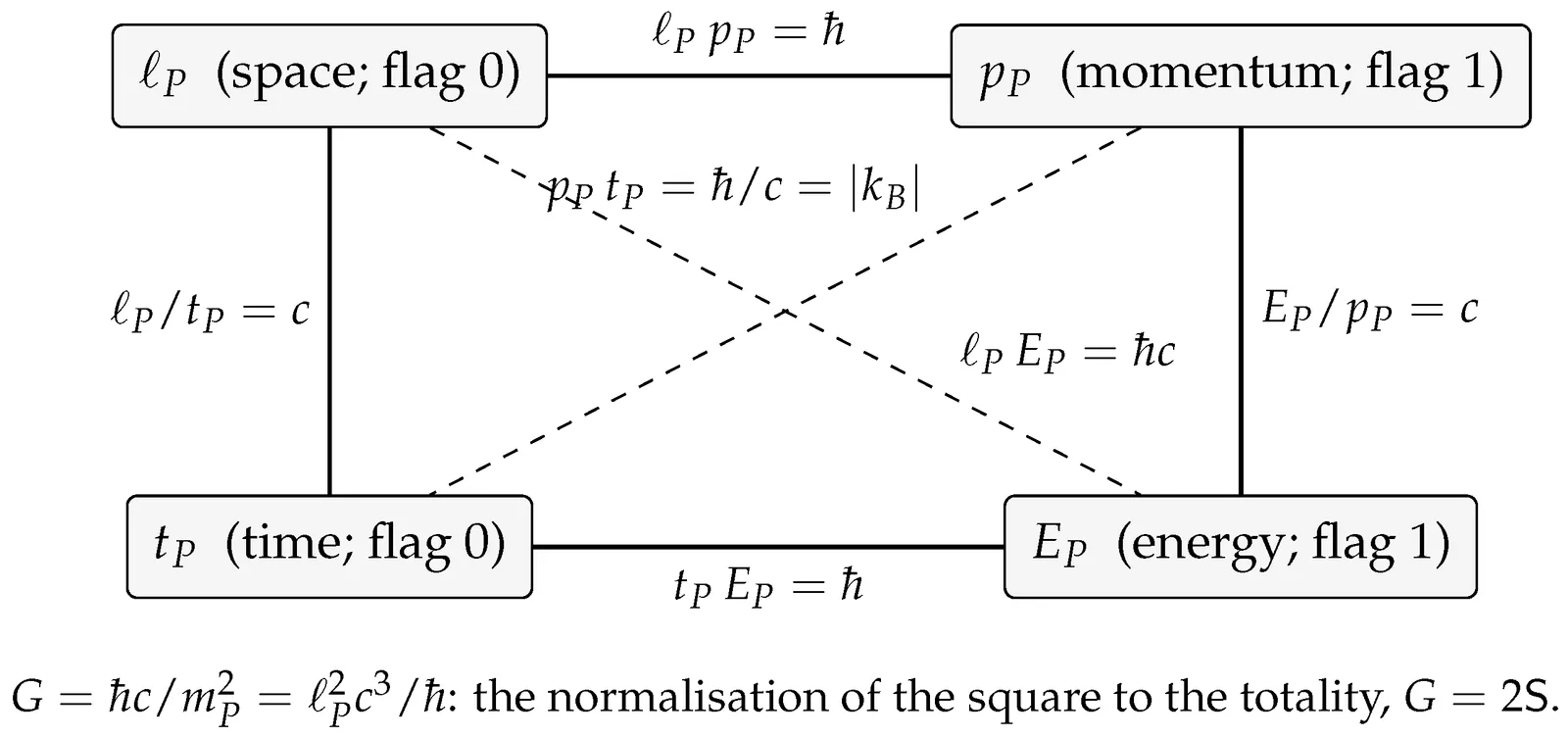
The quartet square. Corners: the four domain horizons, the fundamental units, with their flag positions (0,0,1,1) (Proposition 8); the primal-to-dual passage is the crossed duality $\delta_{C}=i_{D}\;\delta_{S}$, one flag per crossing. Edges: the across-pair ratios both equal *c*; the conjugate-pair products both equal *ℏ* (the two equalities are one identity, Remark 11). Diagonals: the mixed products, $\hbar /c=|k_{B}|$ and ℏc. Beneath the square: the normalisation *G* relating the quartet to the totality’s capacity. Four horizons, four constants, no fifth relation (Theorem 8); each constant’s residue pair the unique solution of its pin (Theorem 9).

**Definition 16**  (The pairing constants)**.** *On the domain square (Figure 4) the quartet admits three classes of invariant cross-domain relation, and each class yields one constant:*

$$c:=\frac{\ell_{P}}{t_{P}}=\frac{E_{P}}{p_{P}}\;(\mathrm{the}\;\mathrm{across}\text{-}\mathrm{pair}\;\mathrm{ratio};\;\mathrm{the}\;\mathrm{second}\;\mathrm{face}\;\mathrm{is}\;\mathrm{Remark}11\mathrm{unwound}),$$

$$\hbar =\ell_{P}\;p_{P}=t_{P}\;E_{P}\;(\mathrm{the}\;\mathrm{conjugate}\text{-}\mathrm{pair}\;\mathrm{products},\;\mathrm{both}\;\mathrm{returning}\;\mathrm{the}\;\mathrm{primitive}\;\mathrm{crossing}\;\mathrm{quantum}),$$

$$p_{P}\;t_{P}=\frac{\hbar }{c},\;\ell_{P}\;E_{P}=\hbar c\;(\mathrm{the}\;\mathrm{mixed}\;\mathrm{products}).$$

*The mixed product $p_{P}t_{P}$ is the magnitude of the Boltzmann constant, $|k_{B}|=\hbar /c$, its partner $\ell_{P}E_{P}=\hbar c$; the residue-face pairs are stated in Theorem 9.*

**Theorem 8**  (Pairing closure: no fifth parameter in the quartet lattice)**.** *The cross-domain relations of the quartet reduce to exactly two independent generators, c and ℏ: the mixed products are their monomials, $p_{P}t_{P}=\hbar /c$ and $\ell_{P}E_{P}=\hbar c$. The Boltzmann constant is the mixed-product face, $|k_{B}|=\hbar /c$: derived, not independent. The one datum not internal to the square is the normalisation of the quartet to the totality,*

$$G:=\frac{\hbar c}{m_{P}^{2}},\;m_{P}:=\frac{p_{P}}{c}=\frac{E_{P}}{c^{2}},$$

*equivalently $G=\ell_{P}^{2}c^{3}/\hbar$. The triples (c,ℏ,G) and $\left(\ell_{P},t_{P},\hbar \right)$ parametrize the same three positive scales bijectively, so the constant set $\left\{c,\hbar ,G,k_{B}\right\}$ follows uniquely from the quartet with exactly three independent members, and no further algebraically independent parameter exists within the quartet relation lattice (ledger:E2).*

**Proof.**  By Definition 15 the quartet generates the free abelian group on $\left\{\ell_{P},t_{P},\hbar \right\}$, of rank three. Direct exponent arithmetic in that group gives $c=\ell_{P}t_{P}^{-1}$, $p_{P}t_{P}=\hbar \;c^{-1}$, $\ell_{P}E_{P}=\hbar \;c$: every pairing of the quartet is a monomial in {c,ℏ}, so the pairing sublattice has rank two. Given *c* and *ℏ*, one free scale remains: $\ell_{P}$, with $t_{P}=\ell_{P}/c$, $p_{P}=\hbar /\ell_{P}$, $E_{P}=\hbar c/\ell_{P}$. The normalisation fixes it, $\ell_{P}^{2}=G\hbar /c^{3}$, recovered by the positive square root: the sublattice 〈c,ℏ,G〉 has index two in the free lattice on $\left\{\ell_{P},t_{P},\hbar \right\}$ (the exponent matrix has determinant −2), so the correspondence of triples is monomial in one direction and closed by the positive root in the other, a bijection of positive-magnitude scale assignments, which is what unit-face scales are. *G*’s non-redundant content is the value it takes on the totality (Theorem 9: G=2S), the one datum the square itself does not carry. Any further invariant combination of the quartet is algebraic over {c,ℏ,G} (a monomial of the index-two sublattice or a positive square root thereof) and introduces no new parameter: within the quartet relation lattice there is no fifth constant. □

**Corollary 3**  (The cancellation identity)**.** *The relation*

$$|k_{B}|\;c=\hbar$$

*is an identity of the quartet: at the unit face,*

$$\left(p_{P}\;t_{P}\right)\cdot \frac{\ell_{P}}{t_{P}}=\ell_{P}\;p_{P},$$

*so the mixed product times the across-pair ratio is the conjugate-pair product exactly. At the residue face the same identity is carried by the one pair {±h}={±ℏ} (Theorem 9); at the unit face h=2πℏ (Section 3), so the h-form of the identity reads $|k_{B}|\;c=h/2\pi$, the frame’s per-cycle rebooking of the per-step quantum.*

**Corollary 4**  (Derived scales)**.** *Mass is derived, not primitive: $m_{P}=p_{P}/c=E_{P}/c^{2}$, in agreement with the mass domain of Section 7. Temperature is closed rather than deferred: the temperature horizon is $\Theta_{P}=E_{P}/\left|k_{B}\right|$ on the positive unit face, and the thermodynamic constant is the mixed-product face of the quartet; no independent thermal unit exists.*

**Corollary 5**  (Degrees of freedom, both faces)**.** *At the unit face the quartet carries one internal identity, leaving three free scales, in bijection with the three independent constants {c,ℏ,G} of Planck’s construction: the arity of classical mechanical bookkeeping, here derived rather than assumed. The classical system’s fourth unit, the thermal dimension, is the torsion-free surrogate of the derived $k_{B}$, exactly as the mass primitive surrogates the flag (Corollary 2): where a derived object is invisible to free-abelian bookkeeping, that bookkeeping adjoins a primitive in its place. At the residue face all four constants are generator-free in the capacity alone: G=2S, $c^{2}=2S+1$, $\hbar^{2}\equiv -1$, $k_{B}^{2}\equiv -2$, so one imported number fixes the quartet’s residue pairs, the constants, and every derived unit, with no freedom within the pin equations (Theorem 9) (ledger:E2).*

**Proposition 8**  (Flag positions of the quartet)**.** *The quartet is not flag-homogeneous: the primal horizons are flag-free,*

$$\left[\ell_{P}\right]=\left[L\right],\;\left[t_{P}\right]=\left[T\right],$$

*while the dual horizons carry one flag,*

$$\left[p_{P}\right]=i_{D}\;{\left[L\right]}^{-1},\;\left[E_{P}\right]=i_{D}\;{\left[T\right]}^{-1},$$

*so the flag positions at the four corners are (0,0,1,1), and every quartet relation closes on them: $\hbar =\ell_{P}p_{P}$ at 0+1=1=[ℏ]; $c=\ell_{P}/t_{P}$ at 0−0=0 and $c=E_{P}/p_{P}$ at 1−1=0, flag-free on both faces; $|k_{B}|=p_{P}t_{P}$ at 1+0=1, a flagged unitful measure; $G=\ell_{P}^{2}c^{3}/\hbar$ at 0−1=−1, the inverse flag. Two maps must be kept distinct here: the chart duality $\delta_{S}:D\mapsto D^{-1}$ of Theorem 6 is flag-free and cannot raise the flag; the primal-to-dual passage is the crossed duality $\delta_{C}=i_{D}\;\delta_{S}$ (Section 5), $\left[p_{P}\right]=\delta_{C}\left[L\right]$, $\left[E_{P}\right]=\delta_{C}\left[T\right]$: the dual horizons are the flag-crossed images of the primal ones (Definition 15). Verified in ledger:E3.*

**Proof.**  All four positions are now by construction: the primal anchorings $\left[\ell_{P}\right]=\left[L\right]$, $\left[t_{P}\right]=\left[T\right]$ are flag-free by Definition 15, and the crossed images carry $\left[p_{P}\right]=\left[\hbar \right]{\left[\ell_{P}\right]}^{-1}=i_{D}{\left[L\right]}^{-1}$, $\left[E_{P}\right]=\left[\hbar \right]{\left[t_{P}\right]}^{-1}=i_{D}{\left[T\right]}^{-1}$, crossing degree one (Definition 14), consistent with Proposition 6 evaluated at the horizons. The construction is confirmed independently by the flag-free geometric conversions of Section 7: $\left[G\hbar /c^{3}\right]={\left[L\right]}^{2}=\left[\ell_{P}^{2}\right]$ and $\left[Gm/c^{3}\right]=\left[T\right]=\left[t_{P}\right]$ place the primal horizons at position zero from the mechanical side. The closure computations are the flag arithmetic of the listed relations. At the count face the same asymmetry reads: the momentum reading is one quarter-turn from the winding count, $p=i_{D}\;k$ in domain form, while no quarter-turn attaches to position or to relative time. □

**Remark 12**  (Two moves, and why there is no *ℏ* of space and time)**.** *The passage from the Subject’s reading to the Carrier’s decomposes into two independent moves, and the two rows of Figure 2 mix them differently. The first is* re-anchoring within the fibre*: trading the frame’s conventional scale anchor (the unit *1*, the chronon step) for the substrate-fixed anchor, the horizon. A length of x conventional units against $x^{\prime }$ Planck lengths is a ratio of two anchors on the* same *chart: a pure number, *Ω*-hard in absolute value, carrying no flag. This is the entire difference between the Subject’s space and time and the Carrier’s. The second move is the* quarter-turn crossing*: the fibre change from a chart to its dual, carrying the flag. Momentum and energy take both moves (their counts re-anchor* and *cross), which is Proposition 8’s (0,0,1,1). Two consequences follow. First, there is no space/time analogue of the Planck constant: no universal constant mediates a coordinate count into a length or a duration, because that passage is a scale convention, not a register crossing. That is exactly why the pairing lattice of Theorem 8 closes on the two generators it has, c the one flag-free relation (a ratio of the two anchored horizons) and ℏ the one flagged relation (the crossing itself). Second, the fundamental data* are *two anchored horizons plus one crossing. Definition 15 takes exactly $\left\{\ell_{P},t_{P},\hbar \right\}$ as primitive, in bijection with {c,ℏ,G}: the same three degrees of freedom as Corollary 5, with the reason for their shape now definitional (ledger:E3).*

### Carrier Instantiation

The constants of this section are Carrier-register residues, and the register is Ω-hard (Section 2): no bounded observer resolves them; their unit-face magnitudes are their only registrable manifestation, which is why the observer’s physics is conducted in units. The Carrier layer is therefore stated once, over unordered sign pairs $\left\{x,-x\right\}\subset F_{\Omega }$. This is the form in which the defining congruences are exact and complete. Nothing in the congruences selects a member. No registration could reveal a selection. The selection itself is a resolved fact of the instantiated totality. What is Ω-hard is its knowability, never its existence. Everything the paper builds on appears above through unit-face magnitudes alone; the statements below are the existence certificate.

**Remark 13**  (Registration)**.** *What a bounded observer can register of the Carrier layer is exactly the pair-inert content: combinations of the residue pairs {x,−x} invariant under every admissible sign assignment, the squares, the pair products, and their ratios. The suite’s inertness check (the Verification paragraph below) is this statement in machine form. The registrable content coincides with what the unit face carries: magnitudes and their ratios, never a sign, never a root selection. The observer-facing dimensionless relations live entirely on that face: the factor 2π between the two presentations of the action quantum is a unit-face Subject-side reading (Remark 14), while at the residue face the two presentations are one pair. No map from a residue pair to an operational magnitude exists or is needed: *Ω*-hardness forbids the former, and the unit face supplies the latter as the pair’s only registrable manifestation (ledger:E5).*

**Theorem 9**  (Uniqueness: the defining congruences, pair form)**.** *On the Carrier chart the four constants satisfy the bounded-height defining relations*

$$2G+1\equiv 0,\;2c^{2}\equiv 1,\;\hbar^{2}\equiv -1,\;k_{B}^{2}\equiv -2\;\left(\mathrm{mod}\;\Omega \right),$$

*and these determine them uniquely at pair level: G=2S=(Ω−1)/2, the Carrier’s half-cycle, exact outright (the congruence is linear); {±c} the root pair of $2^{-1}$; {±ℏ} the root pair of −1; $\left\{\pm k_{B}\right\}$ the root pair of −2; and the linkage*

$$\left\{\pm k_{B}\right\}\;\left\{\pm c\right\}=\left\{\pm \hbar \right\}$$

*holds as a pair identity, derived rather than declared. Given the capacity S, the quartet therefore determines the four constants uniquely at pair level, and conversely (Definition 16, Theorem 8) (ledger:E4).*

**Proof.**  Linearity gives *G* directly: 2G≡−1 has the single solution G=(Ω−1)/2=2S. For the quadratics, $F_{\Omega }^{\times }$ is cyclic, so each of $2^{-1}$, −1, −2 has exactly two square roots when it is a quadratic residue, and Carrier admissibility guarantees all three: Ω=4S+1≡1(mod4) makes −1 a residue unconditionally; S even is exactly Ω≡1(mod8), which makes 2, hence $2^{-1}$, a residue; and −2=(−1)·2 is a residue as a product of residues. Each root pair {x,−x} is a canonical object of $F_{\Omega }$, and pair multiplication is well defined, {±a}{±b}={±ab}, so the linkage is a statement about the pairs, and it is a theorem: ${\left(k_{B}c\right)}^{2}\equiv k_{B}^{2}c^{2}\equiv \left(-2\right)\cdot 2^{-1}\equiv -1$, so the product pair $\left\{\pm k_{B}\right\}\left\{\pm c\right\}$ is the root pair of −1, which is {±ℏ}. No further datum enters. The pairs are unique, and the defining congruences carry no selection. The selection itself is the totality’s, resolved but unregistrable from within. □

**Remark 14**  (The *h*-form)**.** *The root pair of −1 is exactly {ℏ,h}: with h=2πℏ and 2π≡−1 on every shell (Section 3), h≡−ℏ. The two presentations of the action quantum are thus the two members of one Carrier residue pair: one pair, two unit-face presentations, the framework’s h-form pair. The distinction between the members is unit-face and Subject-side (the factor 2π, the half-period doubled); at the Carrier layer there is only the pair. The ± the pairs leave unresolved is not an open parameter, and it is not an ambiguity of the Carrier. The selection is resolved at the totality, on the lab instantiation as on any other. What the pairs leave open is its knowability: no sub-capacity frame distinguishes the members (the window ladder of Section 8). This is the visible form of Ω-hardness. Co-orientation of a Subject frame with a chosen representative (the winding sign read against the frame’s own i) carries content only where registers couple, in the framework’s dynamics and causality layers [38,41]; no statement of this paper depends on it (ledger:E4).*

**Corollary 6**  (Pair consequences)**.** *By $F_{\Omega }$ arithmetic from the defining congruences, each statement pair-well-defined: $G=-c^{2}$ and $G^{2}\equiv 4^{-1}$ (both exact: G is single-valued and the factors enter squared); $\hbar^{4}\equiv 1$; ${\left(k_{B}c\right)}^{2}\equiv -1$; and the monomial $\hbar c\;G^{-1}$ lands in the pair $\left\{\pm k_{B}\right\}$, the residue shadow of the unit-face monomial $m_{P}^{2}=\hbar c/G$, while its horizon face is declared separately below (ledger:E5).*

**Proof.**  $G=-c^{2}$ from 2G≡−1 and $c^{2}\equiv 2^{-1}$; the squares by squaring; ${\left(k_{B}c\right)}^{2}\equiv k_{B}^{2}c^{2}\equiv -1$; and ${\left(\hbar c\;G^{-1}\right)}^{2}\equiv \left(-1\right)\cdot 2^{-1}\cdot 4\equiv -2$, so $\hbar c\;G^{-1}$ lands in the root pair of −2, which is $\left\{\pm k_{B}\right\}$. The defining congruences are true statements in $F_{\Omega }$, and field arithmetic preserves truth; the content of the corollary is the list. The suite verifies every listed statement on both Carriers, under all four admissible representative assignments. □

**The residue assignments (D|R).** The quartet’s two faces are stated separately, and tagged. At the unit face, the Planck chart is the chart in which each horizon is the unit of its own domain; the readings c=1, ℏ=1 of natural-unit practice are this chart’s labels and carry no exact content beyond it. At the residue face, the constants sit in the square-root horizon band of the Carrier: $\hbar^{2}=\Omega -1$ and $k_{B}^{2}=\Omega -2$ as congruences. The mass scale carries two typed faces that must not be composed: its *monomial* face $m_{P}^{2}=\hbar c/G$ lands in the pair $\left\{\pm k_{B}\right\}$ (Corollary 6), while its *horizon* face (the declaration $m_{P}^{2}\;\hat{=}\;\Omega$, the totality itself, the scale a bounded observer meets only as a horizon) is a horizon-scale statement outside $F_{\Omega }$, not a congruence, and enters no arithmetic above. The per-domain assignment is declared, not derived: the space and time horizons hold the chart-unit role of their domains, and the momentum and energy horizons hold the crossing role, one flag above them. These declarations are the realisation content of the section; every relation built on them above is a theorem, and the derived-versus-declared boundary is recorded row by row in Table 2. The realisation stands with two explicit falsifiers: a fifth algebraically independent relation within the quartet lattice (Theorem 8 proves none exists; exhibiting one kills the quartet), and a measured variation of the dimensionless ratios of the constants [21], which would unfix the horizons the assignment anchors. The realisation is staked on the constancy of those ratios rather than assuming it beside the assignment: the totality is one, its cardinality Ω a single fixed value, and the residues the congruences fix are fixed once, with nothing in a timeless torsor to vary them; the dependence of a residue on Ω is not variation.

**Remark 15**  (The observer’s procedure)**.** Ω*-hardness is a demarcation, not a gap: it records where a value is read, and the observer’s procedure is complete without it. An element of $F_{p}$ is not obtained from a measurement by a map. A count registered by the observer (a photon count, a cycle count) is an element of its own register, read through the frame’s canonical representative below p (Definitions 2 and 3), and a coherent registration never reaches the period (the framework’s bound on coherent counts, $\sqrt{p}$, Remark 19); a spectral line is a frequency, a count of phase cycles per chronon step, flag-free; the energy E=hf is its flagged reading (Proposition 6), anchored at the Carrier and registered as every unitful measure is registered, as a ratio to the observer’s own units (Remark 8). What no measurement maps onto is an element of $F_{\Omega }$, and none needs to. What the hardness removes is one expectation only: that the residue G=2S is a number an experiment returns. How an Object’s data enter the Subject’s frame is a question for the framework’s dynamics layer, not for its grammar; the present paper is the grammar those registrations obey. Remark 22 states what the grammar fixes of that layer.*

**Remark 16**  (Measured values and the two faces)**.** *A measured value of a constant, with its uncertainty, is a unit-face datum: a ratio of count systems (Remark 8) registered in the observer’s chart. No uncertainty attaches to the residue face, which is not measured: the exactness of G=2S on the Carrier neither predicts nor contradicts the scatter of a measurement, and the paper models no measurement. Nor does the uncertainty of the imported numeral $S=\left(2.6\pm 0.3\right)\times 10^{122}$ of Remark 1 propagate into any constant: what the capacity fixes, it fixes as relations between residues and as the horizons of the unit face (Corollary 5, Section 9), G=2S among them; the numeral itself enters the paper only as the chart scale of the horizon (Remarks 1 and 2), and no measured value of G is computed from it (Remark 13). Error propagation in the observer’s chart is what it is classically, the chart being untouched. Which constants are measured at all is a convention of the chart. The speed of light is exact by definition since 1983, h and $k_{B}$ since the 2019 revision of the SI, the defining constants of the units, and of the four constants only G remains a measured number: relative standard uncertainty $2.2\times 10^{-5}$ in the 2022 CODATA adjustment, where sixteen determinations are reconciled by an expansion factor of 3.9 [42]; the constants known at the $10^{-10}$ level are dimensionless ratios, frequencies measured against the second, or quantities derived from these through the exact defining constants, the observation that only dimensionless ratios of constants are measurable [20,21] made concrete by the units themselves. With ℏ and c fixed, the number $G=\hbar c/m_{P}^{2}$ in the observer’s units is, up to the exact number ℏc, the Planck mass in the observer’s mass unit, inverted and squared: a torsion balance registers the ratio of the mass horizon to the laboratory’s mass anchor, and its scatter is the state of that registration. Likewise no theory derives the value of h in joule-seconds: fixing it is what defines the kilogram, and a reading of ℏ in any conventional unit is the ratio of the observer’s anchors to the substrate’s, ℏ=1 in the Planck chart. The substrate is a timeless torsor (Definition 1) and carries no noise; the finiteness of the field limits a measurement in one way only, in that the framework’s registrability bound on coherent counts, $\sqrt{p}$, whose exponent reading is the coherence window of Remark 19, bounds what a coherent registration resolves, and nothing finer is claimed.*

*Whether the framework’s constraints bound the uncertainties of the chart has a definite answer. The framework fixes three things about a measured constant. It fixes the constant’s domain ($\left[G\right]=i_{D}^{-1}{\left[L\right]}^{5}{\left[T\right]}^{-3}$, Proposition 11). It fixes the constant’s relations to the others, the bijection of triples and the cancellation identity (Theorems 8 and 9, Corollary 3). And it fixes the constancy of the constant’s dimensionless ratios to the others, on which the realisation is staked (the residue assignments above). It does not fix the scatter of a determination: it models no measurement, and it bounds a registration in one way only. A single registration resolved to one part in N is a coherent count of N (a reading is a count, Remark 8), and coherent counts are bounded at $\sqrt{p}$ (Remark 19). A single coherent registration on a shell of cardinality p therefore resolves no finer than $p^{-1/2}$. The paper fixes no p (Remark 2), so the floor is stated by its bound over the admissible shells: under the encoding condition $p^{2}<\Omega$ it is at least $\Omega^{-1/4}$, of the order of $10^{-31}$ for the chart value of Remark 1 (ledger:G5). That is thirteen orders below the eighteen-digit frequency comparisons of optical clocks [43,44] and twenty-six below the uncertainty of G; in bits, the floor is 102 against the 67 of the finest differential comparison, $7.6\times 10^{-21}$ within one clock species [45], and no measurement has reached it. Conversely, a single coherent registration at relative resolution ε places the observer’s shell at $p\geq \varepsilon^{-2}$, the one informative direction in which the constraint touches the chart. The statistics of repeated registrations is the chart’s own (Remark 20), and inside the floor the model is independent of laboratory noise by construction, as every dimensional analysis is. The physical content of the residue identity G=2S is likewise bounded, and the paper claims no more of it. At the unit face it is the normalisation $G=\hbar c/m_{P}^{2}$ that closes the bijection of triples (Theorem 8), a relation the classical account also has. At the residue face its consequences $G=-c^{2}$ and $G^{2}\equiv 4^{-1}$ (Corollary 6) are consistencies the assignment passes, *Ω*-hard like the identity itself, and two of the residues are realised structurally in the minimal instance: the half-cycle 2S is the antipode of the Carrier ring and the root pair of −1 two of its four cardinals [18]. A number an experiment returns it is not, and the paper does not say it is. What the floor and the constancy of the ratios predict, with their falsifiers, is stated in Section 9 and staked in block P of the ledger (Appendix A).*

**Verification.** The suite separates contingent checks from algebraic corollaries. Contingent, verified exhaustively in exact arithmetic on both Carriers (Ω=233 and the programme’s laboratory Carrier, S=602,140, Ω=2,408,561): each quadratic congruence has exactly two roots, the pair {x,−x}, with existence guaranteed by the residue conditions of Theorem 9; the pair linkage and the *h*-form hold bit-exactly. Corollary, not evidence: $G=-c^{2}$ and $G^{2}\equiv 4^{-1}$ follow algebraically from the defining congruences (Corollary 6) and are checked only as consistency of the implementation. The suite computes with fixed representatives (its internal convention, recorded in its annex) and verifies that the convention is *inert*: every checked identity is pair-well-defined and holds under all four admissible representative assignments, on both Carriers. The unit-face identities of the quartet are checked symbolically, and the minimality of the pair (p,Ω)=(13,233) under the complete admissibility predicate is checked by exhaustive scan (Definitions 1 and 2, Remark 1); Figure 3 (Section 2) displays that minimal pair, every structural residue of both registers in place. The suite accompanies the paper.

## 7. Mechanics and Gravitation as Corollaries

With the flag established and the quartet fixed, the mechanical and gravitational domains follow by algebra.

### 7.1. Speed and the Capacity Budget

The speed domain is the relation between the space-like meridian chart and the time-like phase chart.

**Definition 17**  (Speed domain)**.** *The speed domain is*

$$\left[v\right]:=\left[L\right]{\left[T\right]}^{-1}.$$

This domain statement is independent of any metric or geodesic construction: speed compares the spatial meridian chart with the drive’s phase chart, and the interface expression where speed is defined uses a nonzero chronon-step count, so ${\left[T\right]}^{-1}$ exists. The speed-bound domain is the frame-invariant comparison

$$\left[c\right]=\left[L\right]{\left[T\right]}^{-1};$$

its numerical value depends on the chosen [L] and [T] scale assignments, but the domain relation does not, and its quartet face is the across-pair ratio $c=\ell_{P}/t_{P}=E_{P}/p_{P}$ of Section 6.

The counting face of the bound is a budget statement, with the roles kept separate: the temporal unit is one chronon step of the drive (one application of x↦gx), and the capacity κ is the shell’s information budget, the quarter of the phase cycle, 4κ=p−1, a size with no temporal content. The registrable advance of any chart reading over one quarter of the phase cycle is bounded by that quarter’s count: the budget bounds registration as a count-per-count comparison of the two charts, one chart’s steps against the other’s. On the Carrier register the same bound has its residue face: $c^{2}=2S+1=2^{-1}$, self-dual, a Carrier residue read identically by every frame. The two faces are one statement (the counting face is the observer’s reading of the bound, the residue face the Carrier’s), which also settles the placement question for *c*: *c* is a Carrier residue read by every frame, internal to no single shell and external to none, so no separate “between-shell” and “within-shell” readings of the constant arise.

### 7.2. Mass, Mechanics

The first consequence is the domain of mass.

**Proposition 9**  (Mass domain)**.** *Using the dimensional identity*

$$\left[m\right]=\left[E\right]{\left[v\right]}^{-2},$$

*together with $\left[E\right]=i_{D}\;{\left[T\right]}^{-1}$ (Proposition 6) and $\left[v\right]=\left[L\right]{\left[T\right]}^{-1}$ (Definition 17), one obtains $\left[m\right]=i_{D}\;{\left[L\right]}^{-2}\left[T\right]$.*

**Proof.**  Substitute the energy and speed domains:

$$\left[m\right]=i_{D}{\left[T\right]}^{-1}{\left(\left[L\right]{\left[T\right]}^{-1}\right)}^{-2}=i_{D}{\left[T\right]}^{-1}{\left[L\right]}^{-2}{\left[T\right]}^{2}=i_{D}{\left[L\right]}^{-2}\left[T\right].$$

□

Thus mass is not primitive in this domain system: it is the flagged coupling domain between the temporal phase chart and the inverse square of the additive spatial chart, in agreement with the quartet, where $m_{P}=p_{P}/c=E_{P}/c^{2}$ is derived (Corollary 4). This is a domain identity; it does not by itself assert a microscopic mechanism for mass. The Compton closure is immediate and flag-free: $\left[mc/\hbar \right]={\left[L\right]}^{-1}=\left[k\right]$, the wavenumber of the mass.

The remaining elementary mechanical domains follow similarly. We write $\left[a_{\mathrm{acc}}\right]$ for acceleration to avoid confusing it with the affine shift parameter.

**Proposition 10**  (Basic mechanical domains)**.** *In the FRC-native [L],[T] domain system, $\left[a_{\mathrm{acc}}\right]=\left[L\right]{\left[T\right]}^{-2}$, $\left[F\right]=i_{D}\;{\left[L\right]}^{-1}{\left[T\right]}^{-1}$, $\left[p\right]=i_{D}\;{\left[L\right]}^{-1}$, $\left[S\right]=i_{D}$, $\left[P_{\mathrm{power}}\right]=i_{D}\;{\left[T\right]}^{-2}$, and $\left[P_{\mathrm{pressure}}\right]=i_{D}\;{\left[L\right]}^{-3}{\left[T\right]}^{-1}$.*

**Proof.**  Acceleration is velocity per chronon step:

$$\left[a_{\mathrm{acc}}\right]=\left[v\right]{\left[T\right]}^{-1}=\left[L\right]{\left[T\right]}^{-2}.$$

Force is mass times acceleration:

$$\left[F\right]=\left[m\right]\left[a_{\mathrm{acc}}\right]=\left(i_{D}{\left[L\right]}^{-2}\left[T\right]\right)\left(\left[L\right]{\left[T\right]}^{-2}\right)=i_{D}{\left[L\right]}^{-1}{\left[T\right]}^{-1}.$$

Momentum is mass times velocity:

$$\left[p\right]=\left[m\right]\left[v\right]=\left(i_{D}{\left[L\right]}^{-2}\left[T\right]\right)\left(\left[L\right]{\left[T\right]}^{-1}\right)=i_{D}{\left[L\right]}^{-1},$$

in agreement with p=ℏk and Proposition 6: the flagged wavenumber, from two independent routes. Action is energy times time, the domain $i_{D}$ of Proposition 7. Power is energy per time:

$$\left[P_{\mathrm{power}}\right]=\left[E\right]{\left[T\right]}^{-1}=i_{D}{\left[T\right]}^{-2}.$$

Pressure is force per area:

$$\left[P_{\mathrm{pressure}}\right]=\left[F\right]{\left[L\right]}^{-2}=i_{D}{\left[L\right]}^{-3}{\left[T\right]}^{-1}.$$

□

Action carries the flag itself, $\left[S\right]=\left[h\right]=i_{D}$ (Proposition 7): action, the phase-cycle conversion, and the angular conversion all live in the one torsion domain, and the phase counts formed against them are exactly neutral. Every mechanical quantity of the proposition is its torsion-free shadow times one power of the flag, and the flag cancels in every ratio of equal crossing degree the observer forms between them.

**Remark 17**  (No free exponent)**.** *Every mechanical domain of Proposition 10 is computed from three inputs, [L], [T] and $\left[\hbar \right]=i_{D}$, through the defining relations of mechanics, and the thermal rows of Table 1 through the quartet’s tagged mixed-product assignment (Definition 16, Corollary 7); no exponent is fitted, the one choice inside the flag’s construction, that a register crossing is one quarter of the flag cycle, is argued in Proposition 3, and the declared identifications are tagged in Table 2. The mass domain is over-determined rather than fitted: fixed once by $\left[m\right]=\left[E\right]{\left[v\right]}^{-2}$, it closes on relations it was not fitted to, [m][v]=[p] against the crossing p=ℏk (Proposition 6), $m_{P}=p_{P}/c=E_{P}/c^{2}$ from the horizons alone (Corollary 4), the Compton closure (Section 7) and the geometric conversions (Corollary 8), and [G] from Newton’s law and from the quartet face (Proposition 11), each a consistency of the grading, which is what dimensional homogeneity is. The flag’s cancellation is a theorem with a scope, equal crossing degree (Definition 14), and outside that scope the flag stays: on every unitful measure, on F/a=[m], on $E^{2}$ in the half-period sector (Example 6), on the energy–momentum relation at crossing degree two (Example 3). What the flag adds to classical bookkeeping is stated, and the realisations carrying it stand with their falsifiers (Proposition 5, Section Carrier Instantiation): one action quantum rather than one per conjugate pair (Remarks 11 and 12), mass and temperature derived (Corollary 4), temperature on the acceleration domain (Corollary 7), the arity of classical bookkeeping derived (Corollary 5), and no fifth relation within the quartet lattice (Theorem 8).*

### 7.3. Gravitational Coupling

Newton’s constant translates mass-coupling into geometric length and time scales.

**Proposition 11**  (Domain of Newton’s gravitational constant)**.** *From Newton’s law*

$$F=\frac{Gm_{1}m_{2}}{r^{2}},$$

*the gravitational constant has FRC-native domain $\left[G\right]=i_{D}^{-1}\;{\left[L\right]}^{5}{\left[T\right]}^{-3}$.*

**Proof.**  Newton’s law gives

$$\left[G\right]=\left[F\right]\frac{{\left[r\right]}^{2}}{{\left[m\right]}^{2}}.$$

Using [r]=[L], $\left[F\right]=i_{D}{\left[L\right]}^{-1}{\left[T\right]}^{-1}$, and $\left[m\right]=i_{D}{\left[L\right]}^{-2}\left[T\right]$,

$$\left[G\right]=\left(i_{D}{\left[L\right]}^{-1}{\left[T\right]}^{-1}\right)\frac{{\left[L\right]}^{2}}{i_{D}^{2}{\left[L\right]}^{-4}{\left[T\right]}^{2}}=i_{D}^{-1}{\left[L\right]}^{-1}{\left[T\right]}^{-1}{\left[L\right]}^{2}{\left[L\right]}^{4}{\left[T\right]}^{-2}=i_{D}^{-1}{\left[L\right]}^{5}{\left[T\right]}^{-3}:$$

the coupling that turns two flagged masses into one flagged force carries the inverse flag. The quartet face $G=\hbar c/m_{P}^{2}$ closes on the same domain: $\left[\hbar \right]\left[c\right]{\left[m\right]}^{-2}=i_{D}\left[L\right]{\left[T\right]}^{-1}i_{D}^{-2}{\left[L\right]}^{4}{\left[T\right]}^{-2}=i_{D}^{-1}{\left[L\right]}^{5}{\left[T\right]}^{-3}$. □

On the Carrier register the coupling is the half-cycle itself, $G=2S=-c^{2}$ (Theorem 9): the normalisation of the quartet to the totality, the one constant whose pin is linear and whose value is the capacity read directly.

### 7.4. Temperature

The thermodynamic domain is fixed by the quartet with no further input, and it is not what classical bookkeeping assigns.

**Corollary 7**  (Temperature carries the acceleration domain)**.** *With $\left[k_{B}\right]=\left[p_{P}t_{P}\right]=i_{D}\;{\left[L\right]}^{-1}\left[T\right]$ and the temperature horizon $\Theta_{P}=E_{P}/\left|k_{B}\right|$ (Corollary 4),*

$$\left[\Theta \right]=\left[E\right]\;{\left[k_{B}\right]}^{-1}=\left(i_{D}\;{\left[T\right]}^{-1}\right){\left(i_{D}\;{\left[L\right]}^{-1}\left[T\right]\right)}^{-1}=\left[L\right]\;{\left[T\right]}^{-2}=\left[a_{\mathrm{acc}}\right]:$$

*temperature is flag-free and carries the domain of acceleration (ledger:F3).*

**Proof.**  Direct substitution, the flags cancelling at crossing degree 1−1=0. The classical thermal dimension Θ is a free primitive precisely because torsion-free bookkeeping cannot see $k_{B}$ as the derived mixed face (Corollary 5); once $k_{B}$ is derived, the thermal domain collapses onto a mechanical one. □

**Remark 18**  (The Unruh closure, and scope)**.** *The identification is exactly Unruh-shaped [46,47]: the combination $T_{U}=\hbar a/2\pi c\;k_{B}$ closes flag-free in the algebra,*

$$\left[\frac{\hbar a}{c\;k_{B}}\right]=i_{D}\cdot \left(\left[L\right]{\left[T\right]}^{-2}\right)\cdot \left({\left[L\right]}^{-1}\left[T\right]\right)\cdot \left(i_{D}^{-1}\left[L\right]{\left[T\right]}^{-1}\right)=\left[L\right]{\left[T\right]}^{-2}=\left[\Theta \right],$$

*so the relation that ties temperature to acceleration in horizon thermodynamics closes flag-free in this system: the mixed-product assignment of Definition 16 fixes the temperature domain, and Unruh’s combination is the consistency check that assignment passes. What the present paper does* not *supply is the operational calibration of the FRC temperature reading against thermodynamic temperature; that requires the thermodynamic layer of the programme and is out of scope here. The corpus reads the same domain at the gravitational sector’s acceleration floor $a_{0}=cH_{0}/2\pi$ [48] (ledger:F3).*

Table 1 summarizes the conventional mechanical domains and their native FRC assignments.

Several geometric consequences follow immediately.

**Corollary 8**  (Geometric conversion domains)**.** *The gravitational coupling domain satisfies $\left[G\hbar /c^{3}\right]={\left[L\right]}^{2}$, $\left[Gm/c^{2}\right]=\left[L\right]$, and $\left[Gm/c^{3}\right]=\left[T\right]$. Moreover, if $\rho_{m}$ is mass density and $\rho_{E}$ is energy density, then*

$$\left[G\rho_{m}\right]={\left[T\right]}^{-2},\;\left[\frac{G}{c^{4}}\rho_{E}\right]={\left[L\right]}^{-2}.$$

*Verified in ledger:F2.*

**Proof.**  Since $\left[c\right]=\left[L\right]{\left[T\right]}^{-1}$, we have ${\left[c\right]}^{3}={\left[L\right]}^{3}{\left[T\right]}^{-3}$, and $\left[\hbar \right]=i_{D}$ cancels the inverse flag of [G]. Hence

$$\left[\frac{G\hbar }{c^{3}}\right]=i_{D}^{-1}i_{D}\;{\left[L\right]}^{5}{\left[T\right]}^{-3}{\left[L\right]}^{-3}{\left[T\right]}^{3}={\left[L\right]}^{2}.$$

For a mass *m*,

$$\left[\frac{Gm}{c^{2}}\right]=i_{D}^{-1}i_{D}\;{\left[L\right]}^{5}{\left[T\right]}^{-3}{\left[L\right]}^{-2}\left[T\right]{\left[L\right]}^{-2}{\left[T\right]}^{2}=\left[L\right],\;\left[\frac{Gm}{c^{3}}\right]=\left[T\right].$$

Mass density has domain $\left[\rho_{m}\right]=\left[m\right]{\left[L\right]}^{-3}=i_{D}\;{\left[L\right]}^{-5}\left[T\right]$, so $\left[G\rho_{m}\right]={\left[T\right]}^{-2}$. Energy density has domain $\left[\rho_{E}\right]=\left[E\right]{\left[L\right]}^{-3}=i_{D}\;{\left[L\right]}^{-3}{\left[T\right]}^{-1}$; with

$$\left[\frac{G}{c^{4}}\right]=i_{D}^{-1}{\left[L\right]}^{5}{\left[T\right]}^{-3}{\left[L\right]}^{-4}{\left[T\right]}^{4}=i_{D}^{-1}\left[L\right]\left[T\right],$$

we obtain $\left[\left(G/c^{4}\right)\rho_{E}\right]={\left[L\right]}^{-2}$. □

The last expression has the domain of curvature: the FRC-native domain algebra reproduces the standard dimensional role of the Einstein coupling, energy density converted into curvature by $G/c^{4}$. Every geometric conversion of the corollary is flag-free: the inverse flag of *G* cancels against the flag of the mass, the energy density, or the explicit *ℏ* of the Planck area. This is the general pattern: the flag marks the unitful quantities individually and cancels in every invariant combination formed here (phase counts and geometric conversions alike), which is why the bounded reading of Corollary 2 closes classically without ever registering it.

## 8. Finite Periodicity and Examples

The finite domain system is modular. Since domain labels lie in $D_{p}=C_{p}\times C_{p-1}$ (Definition 9), one has

$${\left[L\right]}^{r+p}{\left[T\right]}^{s}={\left[L\right]}^{r}{\left[T\right]}^{s},\;{\left[L\right]}^{r}{\left[T\right]}^{s+p-1}={\left[L\right]}^{r}{\left[T\right]}^{s}.$$

This is the global finite-periodic structure of the shell. Ordinary nonperiodic exponent bookkeeping is recovered as a local comparison inside a bounded exponent horizon.

**Theorem 10**  (Local recovery of classical exponent bookkeeping)**.** *Let H≥0 be an exponent horizon. Suppose*

4κ>2H.

*Then the finite modular label system $D_{p}$ distinguishes all conventional exponent pairs (r,s) satisfying*

|r|≤H,|s|≤H.

*Verified in ledger:G1.*

**Proof.**  Suppose two conventional exponent pairs (r,s) and $\left(r^{\prime },s^{\prime }\right)$, each with components bounded in absolute value by *H*, have the same image in $D_{p}$. Then $r-r^{\prime }$ is cyclically zero modulo p, and $s-s^{\prime }$ is cyclically zero modulo p−1. But

$$|r-r^{\prime }|\leq 2H,\;|s-s^{\prime }|\leq 2H.$$

Since 4κ>2H, also p=4κ+1>2H. Therefore the only multiples of p or 4κ in this range are zero. Hence $r=r^{\prime }$ and $s=s^{\prime }$. □

Thus, conventional dimensional analysis is the bounded-horizon comparison of the finite modular shell-domain structure.

**Corollary 9**  (Recovery with crossings)**.** *Theorem 10 recovers the flag-free exponent pair; the flagged sector is recovered through the crossing degree (Definition 14). Classical M-L-T bookkeeping embeds injectively into the refined labels $\left(r,s;j\right)\in Z^{3}$ by $M^{u}L^{a}T^{b}\mapsto \left(a-2u,\;b+u;\;u\right)$, the crossing degree j=u carrying the mass exponent exactly. The shell realizes the projection (rmodp,(s+jκ)mod(p−1)) together with the sector jmod4; on the bounded window |r|,|s|≤H with 2H<κ and |j|≤1 (which contains every quantity of Table 1) the realization is faithful. Beyond that window the sector saturates modulo four ($i_{D}^{4}=1$: the fourth power of a flagged quantity returns to sector zero), and it is the integer crossing degree of the bookkeeping, not the sector, that carries the classical mass exponent: classical dimensional analysis is recovered exactly as the Z-lift of the bounded window. The thermal exponent is not an additional recovered generator: classical *Θ*-bookkeeping returns through the derived $k_{B}$ (Corollary 7) (ledger:G1).*

**Proof.**  The embedding is linear and injective on $Z^{3}$ (it is triangular with unit diagonal in the basis (L,T,M)). Realization is a homomorphism by Definition 14. For faithfulness on the window: two triples with |r|,|s|≤H, |j|≤1 and equal realizations satisfy $r\equiv r^{\prime }\;\left(\mathrm{mod}\;p\right)$, hence $r=r^{\prime }$ since 2H<p; and $s+j\kappa \equiv s^{\prime }+j^{\prime }\kappa \;\left(\mathrm{mod}\;4\kappa \right)$ with $|j-j^{\prime }|\leq 2<4$ and $|s-s^{\prime }|\leq 2H<\kappa$, so $(j-j^{\prime })\kappa$ is congruent to an integer of magnitude below κ, forcing $j=j^{\prime }$ and then $s=s^{\prime }$. The saturation statement is 4κ≡0 in $C_{p-1}$. □

**Corollary 10**  (Buckingham’s count)**.** *Let $Q_{1},\ldots ,Q_{N}$ be quantities with integer-lifted labels $\left(r_{\nu },s_{\nu };j_{\nu }\right)\in Z^{3}$, ν=1,…,N (Definition 14), and let $\Lambda \in Z^{N\times 3}$ be the matrix of their labels. A monomial $\prod_{\nu }Q_{\nu }^{k_{\nu }}$ is neutral on the lift (lifted label (0,0;0)) exactly when $k=(k_{\nu })$ satisfies $k^{T}\Lambda =0$, so the independent dimensionless products number N−rankΛ, with rankΛ≤3: Buckingham’s count, the three columns being the coordinates (r,s;j) of the lift, the exponents of [L], [T] and $i_{D}$, the domains of the primitive triple $\left\{\ell_{P},t_{P},\hbar \right\}$ of Definition 15 (Corollary 5). For quantities whose classical dimensions lie in M-L-T the lifted labels are the classical exponents transported by the unimodular embedding of Corollary 9, and the count is the classical one; for thermal quantities the lifted label follows the derived $k_{B}$ (Corollary 7: [Θ]=[a], so Θ/a is neutral here where four-generator bookkeeping gives rank two), and there the two bookkeepings part. Whenever the lifted label of the monomial lies in the window |r|,|s|≤H, 2H<κ, |j|≤1, neutrality on the lift and neutrality of the realized label (Theorem 4) coincide (the pendulum (T,ℓ,g,m): rank 3, one product, $T^{2}g/\ell$; ledger:G4).*

**Proof.**  The lifted label of $\prod_{\nu }Q_{\nu }^{k_{\nu }}$ is $\sum_{\nu }k_{\nu }\left(r_{\nu },s_{\nu };j_{\nu }\right)=k^{T}\Lambda$, by the additivity of Definition 14; it vanishes exactly on the kernel, a free abelian group of rank N−rankΛ. The classical embedding is triangular with unit diagonal, so it preserves rank. The realization map is a homomorphism, injective on the window by Corollary 9, and sends (0,0;0) to the neutral label; so a monomial whose lifted label lies in the window has neutral realized label exactly when its lifted label is zero. □

**Remark 19**  (The window ladder)**.** *Four bounds govern the windows of this paper, and they are distinct. The operative bound is the* coherence window *$H_{\mathrm{coh}}=2\sqrt{\kappa }(I)$: the registrability hierarchy of the framework bounds coherent registration at $\sqrt{p}$, and $2\sqrt{\kappa }=\sqrt{p-1}\approx \sqrt{p}$ is that bound read on the exponent window. Above it sit the algebraic bounds, each used by its own theorem: the recovery window H<κ/2 (Corollary 9), the flag-inaccessibility bound H<κ (Corollary 2), and the covariance bound H<2κ (Theorem 5). For every capacity κ≥17 the ladder is strictly nested,*

$$2\sqrt{\kappa }\;<\;\frac{\kappa }{2}\;<\;\kappa \;<\;2\kappa ,$$

*and for realistic capacities the first inequality is overwhelming: the flag sits quadratically beyond coherent reach, and the near-saturation regime of Remark 7 is never coherently entered. (The toy shell $F_{13}$, κ=3, sits below the nesting threshold and is used here for arithmetic display only.) The hierarchy closes on itself at the top: on the laboratory Carrier read as a shell, $2\sqrt{S}=\sqrt{\Omega -1}\approx \sqrt{\Omega }$; the coherence window of the totality is the saturating-Subject scale (ledger:G2).*

**Remark 20**  (The wrap and the chart)**.** *The finite period is the structure of the observer’s register, not a distortion applied to a continuous signal, and nothing continuous is projected onto the shell: a registered count is the datum, the continuous quantity its chart, and a count cannot exceed the register whose size bounds it. What finiteness does is bound resolution, and the bounds are stated. A registered quantity is a coefficient in $F_{p}$ read in a domain (Definition 10), and it wraps only at p; coherent registration is bounded at $\sqrt{p}$ (Remark 19, the framework’s registrability hierarchy), quadratically below the wrap, so no frequency or wave measurement inside a coherent window reaches the period. The labels, separately, are faithful on the window 4κ>2H (Theorem 10) and the crossing lift on 2H<κ (Corollary 9). Classical dimensional analysis is therefore recovered exactly, on the window and at crossing degree at most one, at every finite p, with no limit taken and none needed; where the finiteness shows is the floor on a single coherent registration (Remark 16) and the framework’s dimensionless discriminators (Section 9), not an in-window measurement. The domain of a derivative is the ratio of domains, $\left[dy/dx\right]=\left[y\right]{\left[x\right]}^{-1}$, by the grading law (Theorem 2); the differential operator itself, the finite difference on the shell, belongs to the shell’s harmonic layer, whose transform on the phase cycle is exact and invertible on the shell [39], and to its dynamics [41]. The statistics of registered counts are not objects of dimensional analysis.*

**Remark 21**  (The electromagnetic domain)**.** *Charge is not a further generator: the domain generators are attached to the charts of the two cyclic actions of the shell (Definition 8, D), and the shell has no third cyclic action to attach one to (Remark 5, T); the ampere, the electrical base unit of the SI, is the torsion-free surrogate of the root below, as the mass and thermal primitives are of the flag and of $k_{B}$ (Corollaries 2, 5). With the Coulomb constant neutral—the Gaussian normalisation, a label declaration as the unit-face labels c=ℏ=1 of the quartet are (Section Carrier Instantiation)—the Coulomb energy $q^{2}/r$ of a charge q gives ${\left[q\right]}^{2}=\left[E\right]\left[L\right]=i_{D}\left[L\right]{\left[T\right]}^{-1}$, on the lift (1,−1;1); equivalently $q_{P}^{2}=\hbar c$, the Planck charge derived from the quartet with no new horizon. The lift has no square root of it (the Gaussian exponents $M^{1/2}L^{3/2}T^{-1}$ embed to $\left(\frac{1}{2},-\frac{1}{2};\frac{1}{2}\right)$), so charge enters as the flag does, an integer degree on the lift, like the crossing degree of Definition 14, realised as a monomial on the shell: 2(r,s)=(1,κ−1) has the meridian solution $r=2^{-1}=2\kappa +1\equiv -2\kappa$, and the phase equation 2s≡κ−1(mod4κ) is solvable iff κ is odd (its left side is even), with exactly two solutions, s=(κ−1)/2 and s+2κ. On a shell of odd capacity $\left[q\right]={\left[L\right]}^{2\kappa +1}{\left[T\right]}^{(\kappa -1)/2}$ up to the element of order two $i_{D}^{2}$, the choice between them a convention, as $i_{D}$ over $i_{D}^{-1}$ in Theorem 7; its space exponent |r|=2κ is the covariance bound (Theorem 5), so charge is a label but never a window label, and on the lift, at the space exponent 2κ+1, its dilation character (Definition 12) is $m^{-(2\kappa +1)}=m^{-1}\left(\frac{m}{p}\right)$, the space generator’s times the quadratic character $m^{2\kappa }=\left(\frac{m}{p}\right)$ (Euler’s criterion): a dilation by a non-square changes the sign of a charge coefficient. On a shell of even capacity no label squares to ${\left[q\right]}^{2}$: the quantities odd in the charge have no domain there, while every even one—$q^{2}$, ${\left[\phi \right]}^{2}=\left[F\right]$, the field energy density $i_{D}{\left[L\right]}^{-3}{\left[T\right]}^{-1}$—is a monomial of [L], [T], $i_{D}$ on every shell; the paper’s shells *13*, *29*, *173*, *229* have odd capacity. The fine-structure ratio $e^{2}/\hbar c$ is neutral by the identity [E][L]=[ℏ][c]: a count, registered in the observer’s chart (Remark 16) and not fixed by the domain algebra; the residue of $q_{P}$ on the Carrier is *Ω*-hard (Section Carrier Instantiation).*

**Example 1**  (The shell of capacity κ=3). *For capacity κ=3, the shell has cardinality p=13 and phase-cycle order 4κ=12. The domain labels lie in $Z_{13}\times Z_{12}$. If the exponent horizon is H=5, then 12>10=2H, so all conventional exponent pairs with entries between −5 and 5 are distinguished exactly by the finite modular labels.*

**Example 2**  (Kinetic energy consistency)**.** *The domain identity $E∼mv^{2}$ is checked by*

$$\left[m\right]{\left[v\right]}^{2}=\left(i_{D}{\left[L\right]}^{-2}\left[T\right]\right)\left({\left[L\right]}^{2}{\left[T\right]}^{-2}\right)=i_{D}{\left[T\right]}^{-1}=\left[E\right].$$

*Thus the usual mechanical expression for energy is compatible with the flagged-reading domain of Proposition 6.*

**Example 3**  (The energy–momentum relation)**.** *The three terms of $E^{2}=p^{2}c^{2}+m^{2}c^{4}$ lie in one fibre: ${\left[E\right]}^{2}=i_{D}^{2}{\left[T\right]}^{-2}$, ${\left(\left[p\right]\left[c\right]\right)}^{2}={\left(i_{D}{\left[L\right]}^{-1}\cdot \left[L\right]{\left[T\right]}^{-1}\right)}^{2}=i_{D}^{2}{\left[T\right]}^{-2}$, and ${\left(\left[m\right]{\left[c\right]}^{2}\right)}^{2}={\left(i_{D}{\left[L\right]}^{-2}\left[T\right]\cdot {\left[L\right]}^{2}{\left[T\right]}^{-2}\right)}^{2}=i_{D}^{2}{\left[T\right]}^{-2}$. The relation is therefore a homogeneous sum (Theorem 3) at crossing degree two, in the half-period sector $i_{D}^{2}={\left[T\right]}^{\pi }$ of Example 6. Its massless case E=pc is Proposition 6’s [E]=[p][c] at crossing degree one: the photon’s momentum is its flagged wavenumber, p=ℏk, from the same crossing as its energy; and $E=mc^{2}$ closes on the energy fibre (Remark 9). The domain algebra fixes the homogeneity and the crossing degree of the relation; the relation itself is a law of the dynamics layer [41] (ledger:G3).*

**Example 4**  (Squaring changes the modular domain)**.** *Let Q=q[L]. Then $Q^{2}=q^{2}{\left[L\right]}^{2}$. The expression $Q+Q^{2}$ is not a homogeneous physical-interface sum, because the first term lies in the length fibre and the second in the area fibre. In FRC this is a modular-domain obstruction: multiplication changes the domain label, and addition remains valid only inside one fixed domain fibre.*

**Example 5**  (Phase exponent neutrality)**.** *The phase exponent in the quantum expression is neutral, $\left[E\right]\left[T\right]{\left[\hbar \right]}^{-1}=\left(i_{D}{\left[T\right]}^{-1}\right)\left(\left[T\right]\right)\;i_{D}^{-1}=1$, as in Proposition 7. The factor ℏ converts conventional angular-action units into phase count, while the quarter-turn label is carried by i.*

**Example 6**  (Flag arithmetic)**.** *The crossing degree of a monomial adds over Z, the sector modulo four. The Planck area combination carries*

$$\underset{\left[G\right]}{\underset{︸}{-1}}+\underset{\left[\hbar \right]}{\underset{︸}{+1}}+\underset{{\left[c\right]}^{-3}}{\underset{︸}{0}}=0,$$

*so $G\hbar /c^{3}$ is flag-free, while G and ℏ separately are not; and $i_{D}^{2}={\left[T\right]}^{\pi }$, the half-period sector, marks a doubly unitful quantity such as $E^{2}$, whose sector returns to zero only at the fourth power while its crossing degree records *4*. No bounded-window power of [L] or [T] ever contributes to the sector, which is the precise sense in which the flag is horizon-inaccessible (Corollary 2).*

**Example 7**  (Schwarzschild length domain)**.** *The expression $Gm/c^{2}$ has domain [L]. This is the dimensional structure behind the Schwarzschild radius: in the FRC-native domain reading, G converts the mass-coupling domain into a geometric length domain.*

**Example 8**  (Gravitational frequency domain)**.** *The expression $Gm/r^{3}$ has domain*

$$\frac{\left(i_{D}^{-1}{\left[L\right]}^{5}{\left[T\right]}^{-3}\right)\left(i_{D}{\left[L\right]}^{-2}\left[T\right]\right)}{{\left[L\right]}^{3}}={\left[T\right]}^{-2},$$

*flag-free, as for every count-valued comparison. Gravitational mass distributed over a spatial scale generates a squared temporal recurrence domain, as in orbital-frequency expressions.*

**The arity of classical bookkeeping.** The recovery theorem explains what classical dimensional analysis sees; the quartet explains how much of it there is. Classical mechanics-plus-thermodynamics bookkeeping runs on three free dimension generators plus one thermal unit. That arity is now derived: the quartet carries one internal identity (Remark 11), leaving three free scales in bijection with {c,ℏ,G}, and the thermal unit is the torsion-free surrogate of the derived $k_{B}$ (Corollary 5). The *M*-*L*-*T* system is the torsion-free shadow of the two-generator-plus-flag system, the mass primitive standing in for the flag (Corollary 2) and the thermal primitive for the derived mixed face, and its count of primitives is the unit-face dimension of the quartet’s relation lattice. Classical bookkeeping is thereby recovered twice over: locally in its exponents (Theorem 10) and globally in its arity.

## 9. Discussion and Conclusions

The construction above resolves the status of physical units and constants in two layers. The upper layer is the quartet. The fundamental physical units are the Carrier’s four horizons: the Planck length, Planck momentum, Planck time, and Planck energy, one per representation domain. The four constants follow uniquely: $c=\ell_{P}/t_{P}=E_{P}/p_{P}$ is the ratio across the two Fourier pairs, $\hbar =\ell_{P}p_{P}=t_{P}E_{P}$ the product within each conjugate pair, $|k_{B}|=\hbar /c=p_{P}t_{P}$ the mixed product, and $G=\hbar c/m_{P}^{2}=2S$ the normalisation of the quartet to the totality. The primitive data are two anchored horizons and one crossing quantum, $\left\{\ell_{P},t_{P},\hbar \right\}$, the dual horizons their crossed images (Definition 15). Exactly three of the four constants are independent: the pairing lattice has rank two (*c*, *ℏ*), $k_{B}$ is the derived mixed face, and *G* closes the correspondence of triples $\left(c,\hbar ,G\right)↔\left(\ell_{P},t_{P},\hbar \right)$ bijectively on positive scales; the relation $|k_{B}|\;c=\hbar$ is a quartet cancellation identity, carried at the residue face by the one pair {±h}={±ℏ} and read at the unit face with h=2πℏ, the frame’s per-cycle rebooking; $m_{P}=p_{P}/c$ and $\Theta_{P}=E_{P}/\left|k_{B}\right|$ are derived, so neither mass nor temperature is a primitive unit; temperature carries the acceleration domain (Corollary 7); and no further algebraically independent relation exists within the quartet lattice (Theorem 8). Each constant’s residue pair is the unique admissible solution of a bounded-height defining congruence, the linkage $\left\{\pm k_{B}\right\}\left\{\pm c\right\}=\left\{\pm \hbar \right\}$ a pair identity derived rather than declared (Theorem 9): given the capacity S, there is no freedom within the defining congruences and the declared assignments, and the ± the pairs leave unresolved is the visible form of Ω-hardness, not an open parameter. The long question of how many constants are fundamental [20] receives a structural answer: none. Four horizons, three pairing relations, one normalisation.

The lower layer is the grammar that carries the quartet. Dimensional analysis is a finite shell-domain calculus: the additive meridian and drive-phase charts of a framed FRC shell generate the modular domain algebra $A_{p}={⨁}_{\left(r,s\right)\in D_{p}}F_{p}{\left[L\right]}^{r}{\left[T\right]}^{s}$ (Definition 10), with physical homogeneity as fibrewise addition and physical invariance as neutrality of the total domain label. The domain of the Planck constant is the unit flag, $\left[h\right]=\left[\hbar \right]=\left[S\right]=i_{D}={\left[T\right]}^{\kappa }$, $i_{D}^{4}=1$, the generator of the unique order-four subgroup the domain lattice itself carries: at the capacity exponent, covariant with the residue quarter-turn, and inexpressible within any sub-capacity exponent window, torsion that free dimension groups do not contain. The chart duality $\delta_{S}:D\mapsto D^{-1}$ generates the four representation domains, and each dual pair carries two tiers of reading: the observer’s flag-free count (wavenumber, frequency) and the unitful measure flagged by $i_{D}$ (momentum, energy), the two tiers being the Subject and Carrier registers, related by the crossed duality $\delta_{C}=i_{D}\;\delta_{S}$, with the flag the register crossing. A unit of measure is thereby exhibited as a constitutively reciprocal relation, counts of one frame per count of another (Remark 8): no single count system carries a unit, and the flag is the domain form of the crossing. Mass is not primitive, $\left[m\right]=i_{D}{\left[L\right]}^{-2}\left[T\right]$, with the flag-free Compton closure [mc/ℏ]=[k]; the gravitational coupling carries the inverse flag, $\left[G\right]=i_{D}^{-1}{\left[L\right]}^{5}{\left[T\right]}^{-3}$, and every geometric conversion (the Planck area, the Schwarzschild length, the Einstein curvature coupling) is flag-free. The flag marks the unitful quantities individually and cancels in every count-valued comparison (every combination that reduces to a pure registrable number), which is why torsion-free bookkeeping never registers it, adjoining instead the free mass primitive of *M*-*L*-*T* as the flag’s surrogate; the arity of that classical system, three generators plus a thermal unit, is the unit-face dimension of the quartet’s relation lattice (Corollary 5).

The two-generator notation is the classical-dimensional interface. In the underlying FRC shell, [L] and [T] are two scale assignments of one framed finite reference-frame structure: the unit 1 anchors the additive meridian scale, and the chronon step of the drive anchors the temporal scale; the origins (spatial 0 and temporal τ) carry no units. The coefficient records a coordinate or phase count inside its assigned modular domain; by itself it is not a physical measurement. The dimensional algebra is the physical-interface projection of one finite reference-frame shell: direct-sum fibres record admissible comparison domains, neutral-domain expressions record invariant profiles, and classical dimensional analysis is recovered locally inside the sub-capacity window (its *L*-*T* bookkeeping directly, its mass exponent through the crossing lift, its thermal exponent as derived), while the finite shell retains its global modular structure.

Table 2 separates the kinds of claim the paper makes. A row tagged T is an algebraic theorem, proved in the text; D a definition; R a realisation, a row stating what a physical term denotes in the substrate, constitutive rather than interpretive, defended by its registered consequences and standing with its explicit falsifier (Section 5 and Section 6 state the three falsifiers); Ω marks content fixed by the totality, inaccessible to any bounded observer. The complete ledger is Appendix A, forty-eight rows, its twenty-eight theorem rows with their witnesses; its block X collects what classical bookkeeping postulates and the construction derives: the arity and the mass primitive (Corollaries 4 and 5), the thermal primitive (Corollary 7), the single action quantum (Theorem 7), the electrical primitive (Remark 21).

The holographic reading places the result in the entropy-bound literature. The bound programmes derive a finite state count behind every horizon and read it as a constraint on continuum theories [13,14,15,16]. On a finite holographic substrate the same count is the primary datum: the capacity S enters this paper once, as the one imported number, and everything unitful follows from it over the stated realisations (Table 2, rows 8 and 15)—the four horizons as its unit-face readings, the four constants as their pairing relations, the flag as the register crossing that the encoding forces. Dimensional analysis, on this reading, is the boundary bookkeeping of a bounded observer: the grammar of what can be registered across a horizon at finite density. The constructed minimal instance [18] carries the same capacity dynamically; the present paper is its unit-structure face.

The paper stakes two predictions of its own, listed with their falsifiers and their present confrontations in block P of the ledger (Appendix A). The first the standard account does not make. No single coherent registration resolves finer than $\Omega^{-1/4}$, of the order of $10^{-31}$, and no coherent count exceeds $\Omega^{1/4}\approx 6\times 10^{30}$ (Remark 16); in bits, the floor is 102 against the 67 of the finest differential frequency comparison [45]. The bounds the standard account places on a clock’s resolution depend on the clock’s mass and on the duration of the registration [49,50]; the mass-independent form, $\delta t\geq {\left(t_{P}^{2}t\right)}^{1/3}$, permits finer resolution than the floor for a coherent registration longer than some twelve minutes, and the floor depends on neither. One coherent registration finer than the floor refutes the holographic reading of Remark 1, against which the registrability bound is read. The second prediction the standard account assumes where the framework derives it. Every dimensionless ratio of the constants is fixed in time and in position, zero drift and zero dipole, because the totality is one and timeless (Definition 1) and, in the framework’s realisation, every constant is a residue fixed on it, the quartet’s by the congruences given S (Theorem 9). A detected variation refutes the realisation; the present bound, $\dot{\alpha }/\alpha =1.8\left(2.5\right)\times 10^{-19}\;{\mathrm{yr}}^{-1}$ [51], is consistent with zero. The framework’s further discriminating numbers are carried by its other papers, and the grammar developed here is the bookkeeping they run on. The vacuum-fraction octant consumes this paper’s angle–count dictionary (2π the full cycle, h=2πℏ read in the chart; Section 3, Remark 14) as a member of its realisation set: $\Omega_{\Lambda }=\tanh^{2}\left(3\pi /8\right)=0.6837$, against the fitted 0.685±0.007, a 0.19σ landing [52]. The other papers’ numbers are the strong-field departures of the gravitational sector, the black-hole shadow +4.6%, the ringdown frequency −4.4%, the ISCO −6.9% [48], and the flavour-sector spectrum [53]. The falsifiers of Section 5 and Section 6 guard the grammar; block P states what the paper itself predicts.

**Remark 22**  (The grammar and the dynamics layer)**.** *The separation between the grammar of the registrations and the dynamics of the registered systems is the one dimensional analysis has always had with the equations of motion. Buckingham’s count constrains every law without being one, and an empirically closed law that violated the count would refute it; this paper states its own falsifiers for the same purpose (Section 5 and Section 6). The grammar constrains the framework’s dynamics layer in three ways, each a theorem above, the second over the realisation of Proposition 5. First, every law of that layer is a homogeneous sum in $A_{p}$ at a fixed crossing degree (Theorem 3, Definition 14; Example 3 for the dispersion relation), so a law that adds across fibres is excluded, as dimensional homogeneity excludes it classically. Second, every phase factor counts action in one quantum, ℏ on the flag, the same for E=hf and for p=ℏk (Theorem 7, Propositions 5–7), written in the quarter-turn i of the shell’s own phase cycle (Section 3, Example 5). Third, the labels of that layer read classically on the window and at crossing degree at most one (Theorem 10, Corollary 9), so no law of the dynamics layer departs from its classical dimensional form inside the window. Where the finiteness shows is the floor on a single coherent registration (block P) and the framework’s dimensionless discriminators (Remark 20, the paragraph above), not an in-window label. A datum has the same form in both layers. A registered count, a photon count or a length reading, is a coefficient in $F_{p}$ read in the domain of the chart it was counted on, meridian steps in [L] and drive steps in [T] (Definitions 4, 8, 10). The dynamics layer advances it by the drive, the framework’s reading of the observer’s time (Remark 5). The minimal instance of that layer exists and is executed. On the pair (13,233) the objectless phase sector of quantum dynamics is constructed complete, the Schrödinger evolution as the rigid rotation of the holographic encoding of the observer’s sky and the Dirac flow as the gauge deformation finiteness forces [18]. Every structural theorem of that instance is proved at general p and machine-verified in integer arithmetic end to end; the Schrödinger and Dirac dynamics on a general shell are [41]. No Object is registered in that instance: it is subject to the constraints above on the same pair, and the protocol of a registration is what Remark 15 leaves to the dynamics layer. The present paper states the constraints once, for every shell, and claims nothing of the dynamics beyond them.*

The scope of this paper remains bounded. It proves the modular [L],[T]-domain algebra, establishes the flag, states the quartet, and derives the constants and the basic mechanical and gravitational domains. The electromagnetic domain is fixed by Remark 21, the charge a monomial of the two generators on the shells of odd capacity and the fine-structure ratio a neutral count the domain algebra does not fix; the temperature domain is closed at the level of its horizon, its constant, and its domain (the acceleration domain, with the Unruh combination closing flag-free as the assignment’s consistency check, Corollary 7, Remark 18), while thermodynamics itself is not developed; and the dynamics of the domains fixed here belong to the framework’s other layers: the Schrödinger and Dirac dynamics on the shell with their spinor structure [41], the gravitational sector’s dynamics and its strong-field departures [48], and the finite phase propagation of quantum dynamics, whose minimal instance is constructed and machine-verified as a model existence proof in [18]. No continuum limit is taken anywhere above: classical dimensional analysis is recovered exactly at every finite p, on the window and at crossing degree at most one (Theorem 10, Corollary 9), and the continuum is the observer’s local reading of that window (Remark 20). Within that scope the main result is compact: physical unit readings are modular domains of the FRC reference-frame shell; the fundamental units are the Carrier’s four domain horizons; and the four physical constants are the unique pairing relations and normalisation of that quartet—one imported capacity, four horizons, four constants, and no freedom in between.

During the preparation of this manuscript, the author used Anthropic Claude Opus 4.6 and Fable 5 for the purposes of literature review, proofreading, as well as development, numeric verification and formalization of technical claims. The author has reviewed and edited the output and takes full responsibility for the content of this publication.

## Figures and Tables

**Table 1:** Conventional mechanical domains and their FRC-native domain assignments.

Conventional Domain	Meaning	FRC-Native Domain
[h]=[ℏ]=[S]	action; the unit flag, order four, unit-free	$i_{D}$
[E]	energy: flagged frequency; horizon $E_{P}$	$i_{D}\;{\left[T\right]}^{-1}$
[M]	mass; derived, $m_{P}=p_{P}/c$	$i_{D}\;{\left[L\right]}^{-2}\left[T\right]$
[p]	momentum: flagged wavenumber; horizon $p_{P}$	$i_{D}\;{\left[L\right]}^{-1}$
[G]	gravitational coupling; G=2S	$i_{D}^{-1}\;{\left[L\right]}^{5}{\left[T\right]}^{-3}$
$\left[k_{B}\right]$	thermodynamic conversion; derived, $|k_{B}|=\hbar /c$	$i_{D}\;{\left[L\right]}^{-1}\left[T\right]$
[Θ]	temperature: the acceleration domain; $\Theta_{P}=E_{P}/\left|k_{B}\right|$	$\left[L\right]{\left[T\right]}^{-2}$

**Table 2:** Status of the headline claims: T theorem; D definition; R realisation; I import; Ω Ω-hard.

	Claim	Status
1	Modular unit-domain group and grading (Theorems 1 and 2)	T
2	Fibrewise addition and neutral-domain criterion (Theorems 3 and 4)	T
3	Reframing action; covariance; the p−1 period forced, the p refinement declared, its transport theorem (Theorem 5, Remark 3, Proposition 4)	T | D
4	Local recovery of classical bookkeeping, with crossing degree (Theorem 10, Corollaries 9 and 10)	T
5	The two charts of one framed shell as the two domain generators [L],[T]	D
6	The internal flag: $i_{D}={\left[T\right]}^{\kappa }$ unique of order four; no flag of space (Theorems 6 and 7, Corollary 1)	T
7	$\left[h\right]=\left[\hbar \right]=i_{D}$, the anchoring (realisation content); $\left[E\right]=i_{D}\left[f\right]$, $\left[p\right]=i_{D}\left[k\right]$ and horizon-inaccessibility, theorems over them; the order-four premise argued by the operator-cycle lift (Propositions 3, 5, 6, Corollary 2)	R | T
8	The quartet: primitive triple $\left\{\ell_{P},t_{P},\hbar \right\}$; dual horizons crossed images (Definition 15)	R
9	One crossing, one action: $\ell_{P}p_{P}=t_{P}E_{P}$; domain completeness—direct readings of the quartet definition (Remarks 11 and 10)	T | R
10	Pairing closure: no additional algebraically independent scale parameter within the quartet relation lattice (Theorem 8)	T
11	Root pairs canonical and the linkage a pair identity, given the defining congruences (Theorem 9); the congruences themselves the constants’ Carrier assignment (row 15)	T | D
12	Pair consequences; the representative convention inert (Corollary 6)	T
13	Flag positions (0,0,1,1) derived; no *ℏ* of space/time (Proposition 8)	T
14	Unsigned cancellation identity $|k_{B}|c=\hbar$ (Corollary 3; pair form: rows 11–12); $m_{P}$, $\Theta_{P}$ derived; $\left[\Theta \right]=\left[L\right]{\left[T\right]}^{-2}$ (Corollary 7)	T
15	The horizon declarations and per-domain assignment; absolute unit values	D | Ω
16	Mass derived, $\left[m\right]=i_{D}{\left[L\right]}^{-2}\left[T\right]$; $\left[G\right]=i_{D}^{-1}{\left[L\right]}^{5}{\left[T\right]}^{-3}$; count-valued comparisons flag-free	T
17	Two predictions with their falsifiers: the resolution floor $\Omega^{-1/4}$ on single coherent registration; the constancy of the dimensionless ratios (Remark 16, Section 9, block P)	T

## Data Availability

No new empirical data were created or analysed in this study. The numeric verification suite for every exact claim is public: https://finitering.space/10-dimensions/ (accessed on 7 October 2026).

## Appendix A. Predicate Ledger, Machine Verification and Formalisation

The main claims of the paper, listed in Table A1, are machine verified in Python and formalised in Lean 4:

27	Python checks (py): finitering.space/src/10-dimensions/ (accessed on 7 October 2026)
24	Lean core axiom-free proofs (lean): finitering.space/lean/core/Dimensions.html (accessed on 7 October 2026)
22	Lean Mathlib-based proofs (leanML): finitering.space/lean/Dimensions.html (accessed on 7 October 2026)

Blocks follow the paper structure:

A	Inputs,
B	The registers and the labels (Section 2 and Section 3),
C	The modular domain algebra (Section 4),
D	The four domains and the unit flag (Section 5),
E	The quartet and its Carrier instantiation (Section 6),
F	Mechanics, gravitation, temperature and the electromagnetic domain (Section 7, Remark 21),
G	Periodicity, windows, the worked examples and the resolution floor (Section 8, Remark 16),
P	The paper’s predictions, each with its falsifier and its present confrontation (Remark 16, Section 9),
X	What the construction explains (Corollaries 4, 5, 7, Theorem 7, Remark 21).

The structures exercised are the toy shell $F_{13}$ (κ=3), the shells p=29 and p=229, the laboratory-scale shell p=173, and both instantiated Carriers, Ω=233 (S=58) and the laboratory Carrier Ω=2,408,561 (S=602,140); the resolution floor and its prediction (G5, P1) are decided on the chart value Ω=4S+1, $S=2.6\times 10^{122}$, of Remark 1, the constancy prediction (P2) on the imported drift bound. Table 2 is the condensed form of this ledger; the rows here are the statements it summarises. The corpus master ledger carries D7 under its row D7, F3 under its row C12, E9 and G2 under its row C13, E4 under its row C8, and E5 under its row B10.

Status tags:

I	**Import.** A standard result, a corpus result, or a convention used here without reproof.
D	**Definition.** A naming, a declared dictionary, or a set-up move, carrying no empirical content.
T	**Theorem.** Derived within this paper from the rows above it, machine-verified in exact arithmetic by the named witness where a witness is named.
R	**Realisation.** States what a physical term denotes on the substrate: constitutive, not interpretive, and carrying its falsifier.
Ω	**Ω-hard.** A value fixed by the totality, in no bounded observer’s window; a demarcation of where the value is read, not an open item.

**Table A1:** The predicate ledger.

Row	Description	Status	Source
**A. Inputs: imported, not derived here**			
A1	The framed substrate: the Subject shell $F_{p}\left(\tau ;0,1,g\right)$ with p=4κ+1, the derived quarter-turn $i=-g^{\kappa }$, the phase cycle 4κ=2π=p−1, the framed-complex labels; the Carrier C of cardinality Ω=4S+1 with its admissibility congruences; the realisation doctrine (a row stating what a physical term denotes, defended by its consequences and killed by its falsifier).	I	[18,24,25,38]
A2	The holographic principle as the count: the capacity S is the de Sitter entropy, the finite number of physical states behind the observer’s horizon, the one imported number of the paper.	I	[13,14,15,16]
A3	The shell Fourier structure: on $F_{p}^{\Phi }$, $\Phi =C_{p-1}$, the matrix $W_{jk}=g^{jk}$, the normalized operator F=iW with $F^{2}=J$, $F^{4}=I$, and the fractional family with its cardinal skeleton {0,κ,2κ,3κ}.	I	[39]; Remark 6
A4	Classical dimensional analysis and the Planck units: the Π-theorem, the free-abelian exponent group of *M*-*L*-*T*, Planck’s construction of the natural units from {c,ℏ,G}, and the observation that only dimensionless ratios of constants are measurable.	I	[2,3,4,19,20,21]
A5	The registrability hierarchy of the framework: coherent registration bounded at $\sqrt{p}$, read on the exponent lattice as the coherence window $2\sqrt{\kappa }=\sqrt{p-1}$; the finite-field algebra of square classes and cyclic groups used throughout.	I	[26,48]; Remark 19
A6	The Unruh and Hawking temperature forms, $T_{U}=\hbar a/2\pi c\;k_{B}$, as the shape the temperature domain closes on.	I	[46,47]
**B. The registers and the labels**			
B1	The Carrier: the finite complete totality, a timeless torsor of Ω attribute-free points, its cardinality its only absolute characteristic, its reframings x↦ax+b automorphisms; the Subject: a bounded observer, an embedded prime shell of capacity κ with frame data $F_{p}\left(\tau ;0,1,g\right)$, $p^{2}<\Omega$; the two definitions together the paper’s reading of the holographic principle.	D	Definitions 1 and 2, Remarks 1 and 2
B2	The Object: an observed system registered relative to the Subject’s frame, every entry a Subject residue, (t;q,k,v)—relative time, position, winding, rate.	D	Definition 3
B3	Units as scale assignments: the additive meridian action $\left(F_{p},+\right)$ and the multiplicative phase action $F_{p}^{\times }$ are unit-free coordinate actions, a physical unit a scale assignment to a frame chart of them, a measure a coordinate reading relative to the assigned scale; a physical-interface reading is a coefficient with the domain label it is read in, the two periods typing the label exponents; a change of scale assignment leaves $\mathrm{card}(F_{p})=p$ and $\mathrm{card}(F_{p}^{\times })=p-1$ untouched.	D	Definitions 4 and 5, Properties 1
B4	The residue phase label $g^{k}$ with the cardinal packet $1,g^{\kappa },g^{2\kappa },g^{3\kappa }$; the framed-complex label space $C_{p}=F_{p}\times F_{p}$ with its p-to-one evaluation; i the finite shell quarter-turn label.	D	Definitions 6 and 7, Properties 2
**C. The modular domain algebra**			
C1	The two domain generators: [L] attached to the additive meridian chart, anchored on the unit 1; [T] attached to the drive’s phase chart, anchored on the chronon step; the origins carry no units. The modular labels $U_{r,s}={\left[L\right]}^{r}{\left[T\right]}^{s}$ on $D_{p}=C_{p}\times C_{p-1}$, the homogeneous quantity algebra $A_{p}=⨁F_{p}U_{r,s}$, the domain ${\mathrm{dom}}_{p}\left(Q\right)$ of a homogeneous quantity.	D	Definitions 8–11
C2	The modular unit-domain group and the grading: $\left\{U_{r,s}\right\}$ is a finite abelian group isomorphic to $D_{p}$ under $U_{r,s}U_{r^{\prime },s^{\prime }}=U_{r+r^{\prime },s+s^{\prime }}$, and $A_{p}$ is $D_{p}$-graded, $A_{p}^{\left(r,s\right)}A_{p}^{\left(r^{\prime },s^{\prime }\right)}\subseteq A_{p}^{\left(r+r^{\prime },s+s^{\prime }\right)}$.	T	Theorems 1 and 2 py:C2, lean:C2, leanML:C2
C3	Fibrewise addition and the neutral-domain criterion: a sum of homogeneous quantities is homogeneous exactly when its nonzero summands share a domain, the fibre components adding separately; a monomial $\prod Q_{j}^{k_{j}}$ has domain $\sum k_{j}\left(r_{j},s_{j}\right)$ and is a neutral-domain invariant iff that sum is (0,0) in $D_{p}$.	T	Theorems 3 and 4 py:C3, lean:C3, leanML:C3
C4	Admissible reframings: the triples (a,m,ϵ)—translation of the spatial origin, dilation of the unit, re-presentation of the drive $g\mapsto g^{\epsilon }$, $\epsilon \in Z_{p-1}^{\times }$—and the two actions they induce on labels.	D	Definition 12
C5	Window covariance: on a bounded window |r|,|s|≤H<2κ in the flag-free sector the labels are covariant under every admissible reframing; the bound is sharp (the label (0,π) is pushforward-invariant yet non-neutral); the naive character is ill-defined on the modular projection and the would-be temporal character fails composition ($5^{2}\equiv 1$ in $Z_{12}^{\times }$); on the realized lattice the invariant labels are exactly {0,2κ}; the σ-twisted action is equivariant by full sweep on p=13 and 229; the flagged label (0,0;1) moves under ϵ=−1.	T	Theorem 5 py:C5, leanML:C5
C6	What the action forces and what is declared: the time-exponent period p−1 is forced by the pushforward action of $Z_{p-1}^{\times }$, the space-exponent refinement to period p is declared and carried by the transport theorem (D2); classical temporal unit change is nominal re-assignment within a fixed presentation, the chronon atomic.	T	Remarks 3–5, Proposition 4 lean:C6, leanML:C6
**D. The four domains and the unit flag**			
D1	Chart duality: $\left[f\right]={\left[T\right]}^{-1}$; $\delta_{S}:D\mapsto D^{-1}$ is an involution of the domain group whose orbit on the generators is the four-domain structure (space, momentum, time, energy); $\delta_{C}$ the second involution, carrying [L]↦[p], [T]↦[E].	T	Definition 13, Theorem 6 py:D1, lean:D1, leanML:D1
D2	The internal flag: $D_{p}$ contains exactly one subgroup of order four, $\langle {\left[T\right]}^{\kappa }\rangle$, entirely in the time-exponent factor; $i_{D}:={\left[T\right]}^{\kappa }$, $i_{D}^{4}=1$, $i_{D}^{2}={\left[T\right]}^{\pi }$; no flag of space ($Z_{p}$ has no element of order four); the two periods interfere, ${\left(\left[L\right]{\left[T\right]}^{\kappa }\right)}^{p}=i_{D}$, on three shells.	T	Theorem 7, Corollary 1, Proposition 4 py:D2, lean:D2, leanML:D2
D3	The lift: the flag records the quarter the chart duality forgets—on charts the cardinal skeleton acts as smod2 (*F* exchanges the conjugate charts, *J* fixes them), on labels $s\mapsto i_{D}^{s}$ is an isomorphism $Z_{4}\to \langle i_{D}\rangle$ whose quotient by {0,2κ} returns the chart action; on the Carrier $\hbar^{2}=-1$, $\hbar^{4}=1$, $\hbar^{2}\neq 1$, the crossing quantum of order four, never two. Shells (13,2), (173,3); Carriers 233, 2408561.	T	Proposition 3 py:D3, lean:D3, leanML:D3
D4	The crossing degree: the full label (r,s;j), j∈Z the signed number of register crossings, realized as $U_{r,\;s+j\kappa }$ with the sector jmod4.	D	Definition 14
D5	The unit flag: the Planck constants carry the flag, $\left[h\right]=\left[\hbar \right]=i_{D}$—the anchoring of the action quantum to the order-four flag, the realisation content of the paper. Falsifier: one empirically closed expression that adds an observer count to a unitful measure without the conversion quantum (an energy to a frequency, a momentum to a wavenumber).	R	Proposition 5, Section 5
D6	The flagged readings over the counts: $\left[E\right]=\left[h\right]\left[f\right]=i_{D}{\left[T\right]}^{-1}$, $\left[p\right]=\left[\hbar \right]\left[k\right]=i_{D}{\left[L\right]}^{-1}$, one quantity per pair, the flagged reading one capacity step above its count; $\left[S\right]=\left[E\right]\left[T\right]=i_{D}$ and every phase count neutral, $\left[E\right]\left[T\right]{\left[\hbar \right]}^{-1}=1$; the absolute unit is horizon-inaccessible (no monomial of a window H≪κ expresses the flag) and becomes visible at κ≤H<2κ.	T	Propositions 6 and 7, Corollary 2, Remark 7 py:D6, lean:D6, leanML:D6
D7	A unit of measure is a reciprocal relation between two count systems, counts of one frame per count of another; the unit flag is its domain form, the register crossing made algebraic; the Planck relation the unique interface introducing energy without a mass primitive. Falsifier: an absolute unit value registered by an embedded observer, or a unitful measure carrying no cross-frame reciprocal.	R	Remarks 8 and 9
**E. The quartet and its Carrier instantiation**			
E1	The quartet: the primitive triple $\left\{\ell_{P},t_{P},\hbar \right\}$—the two anchored horizons, flag-free re-anchorings of the two primal charts, $\left[\ell_{P}\right]=\left[L\right]$, $\left[t_{P}\right]=\left[T\right]$, and the crossing quantum—with the dual horizons their crossed images, $p_{P}=\hbar /\ell_{P}$, $E_{P}=\hbar /t_{P}$; the four horizons occupy the four domains, flag positions (0,0,1,1); the pairing constants $c=\ell_{P}/t_{P}=E_{P}/p_{P}$, $\hbar =\ell_{P}p_{P}=t_{P}E_{P}$, $|k_{B}|=p_{P}t_{P}$, and *G* the one datum not internal to the square. The fundamental units are the Carrier’s horizons. Falsifiers: a fifth algebraically independent relation within the quartet lattice; a measured variation of the dimensionless ratios of the constants.	R	Definition 15, Remark 10, Definition 16, Section 6
E2	One crossing, one action, and pairing closure: $\ell_{P}p_{P}=t_{P}E_{P}=\hbar$ (the two faces of *c* and *ℏ* differ by the single relation ℓp=tE); the cross-domain relations reduce to the two generators *c*, *ℏ*, the mixed products their monomials, $|k_{B}|=\hbar /c$ derived; the relation lattice over $\left\{\ell_{P},t_{P},\hbar \right\}$ has rank two and $k_{B}$, $\ell_{P}E_{P}$ add nothing; 4−1=3 free scales in bijection with {c,ℏ,G}, the classical arity derived.	T	Remark 11, Theorem 8, Corollary 5 py:E2, leanML:E2
E3	The cancellation identity $|k_{B}|c=\hbar$ at the unit face, $\left(p_{P}t_{P}\right)\left(\ell_{P}/t_{P}\right)=\ell_{P}p_{P}$; mass derived, $m_{P}=p_{P}/c=E_{P}/c^{2}$; the temperature horizon $\Theta_{P}=E_{P}/\left|k_{B}\right|$; the flag positions (0,0,1,1) and the closure of every quartet relation on the flag component; no *ℏ* of space and time.	T	Corollaries 3 and 4, Properties 8, Remark 12 py:E3, leanML:E3
E4	The defining congruences, pair form: on the Carrier chart 2G+1≡0, $2c^{2}\equiv 1$, $\hbar^{2}\equiv -1$, $k_{B}^{2}\equiv -2\;\left(\mathrm{mod}\;\Omega \right)$ determine the constants uniquely at pair level—G=2S exact, the quadratics each with exactly two roots {x,−x} (admissibility guarantees the residues: Ω≡1mod4, S even for 2, hence −2)—and the linkage $\left\{\pm k_{B}\right\}\left\{\pm c\right\}=\left\{\pm \hbar \right\}$ is derived at pair level; the root pair of −1 is {ℏ,h} with h=2πℏ≡−ℏ. Exhaustive on both Carriers.	T	Theorem 9, Remark 14 py:E4, lean:E4, leanML:E4
E5	Pair consequences and representative inertness: $G=-c^{2}$, $G^{2}\equiv 4^{-1}$, $\hbar^{4}\equiv 1$, ${\left(k_{B}c\right)}^{2}\equiv -1$, and $\hbar cG^{-1}$ lands in $\left\{\pm k_{B}\right\}$; of the eight sign assignments exactly the four with $\sigma_{\hbar }=\sigma_{c}\sigma_{k}$ are admissible, a ${\left(Z/2\right)}^{2}$, every identity holding on each and the *ℏ*-flip relabelling within {ℏ,h}; what a bounded observer registers is exactly the pair-inert content.	T	Corollary 6, Remark 13 py:E5, lean:E5, leanML:E5
E6	The residue assignments: the unit face (the Planck chart, each horizon the unit of its domain; c=1, ℏ=1 its labels) and the residue face (the square-root horizon band, $\hbar^{2}=\Omega -1$, $k_{B}^{2}=\Omega -2$); the mass scale’s two typed faces, the monomial $m_{P}^{2}=\hbar c/G$ in $\left\{\pm k_{B}\right\}$ and the horizon declaration $m_{P}^{2}\;\hat{=}\;\Omega$, never composed.	D	Section Carrier Instantiation (the residue assignments)
E7	The Carrier register is Ω-hard: no bounded observer resolves the constants’ residues; the selection of a member in each pair {x,−x} is a resolved fact of the instantiated totality, knowable to no sub-capacity frame (the window ladder, G2); the absolute unit values are read at the totality and their unit-face magnitudes are their only registrable manifestation.	Ω	Section Carrier Instantiation, Remarks 14–16
E8	The two moves from the Subject’s reading to the Carrier’s: re-anchoring within the fibre (the unit and the chronon step traded for the substrate-fixed marks) and the register crossing (the flag); the two rows of the four-domain figure mix them differently.	D	Remark 12
E9	The minimal admissible pair: under the programme’s admissibility predicate (p=4κ+1 prime, κ>1; Ω=4S+1 prime, S even, S≡1mod3; $p^{2}<\Omega$) the smallest instance is (p,Ω)=(13,233), by exhaustive scan, with the counterfactuals: dropping the mod-3 clause admits (13,193), dropping κ>1 admits (5,41), κ=2 gives the composite 9.	T	Definitions 1 and 2, Remark 1, Section Carrier Instantiation py:E9, lean:E9
**F. Mechanics, gravitation, temperature and the electromagnetic domain**			
F1	The speed domain $\left[v\right]=\left[L\right]{\left[T\right]}^{-1}$ and the capacity budget it is read against.	D	Definition 17, Section 7.1
F2	The mechanical and gravitational domains: $\left[m\right]=i_{D}{\left[L\right]}^{-2}\left[T\right]$; $\left[a\right]=\left[L\right]{\left[T\right]}^{-2}$, $\left[F\right]=i_{D}{\left[L\right]}^{-1}{\left[T\right]}^{-1}$, $\left[p\right]=i_{D}{\left[L\right]}^{-1}$, $\left[S\right]=i_{D}$, $\left[P\right]=i_{D}{\left[T\right]}^{-2}$, $\left[\mathrm{pressure}\right]=i_{D}{\left[L\right]}^{-3}{\left[T\right]}^{-1}$; $\left[G\right]=i_{D}^{-1}{\left[L\right]}^{5}{\left[T\right]}^{-3}$; the geometric conversions $\left[G\hbar /c^{3}\right]={\left[L\right]}^{2}$, $\left[Gm/c^{2}\right]=\left[L\right]$, $\left[Gm/c^{3}\right]=\left[T\right]$, $\left[G\rho_{m}\right]={\left[T\right]}^{-2}$; mass derived, not primitive.	T	Properties 9–11, Corollary 8 py:F2, lean:F2, leanML:F2
F3	Temperature carries the acceleration domain: $\left[\Theta \right]=\left[E\right]{\left[k_{B}\right]}^{-1}=\left[L\right]{\left[T\right]}^{-2}=\left[a\right]$, flag-free; the Unruh combination $\hbar a/c\;k_{B}$ closes flag-free in the algebra; neither mass nor temperature is primitive, the classical *M*-*L*-*T*-plus-thermal system the torsion-free shadow of two generators plus the flag.	T	Corollary 7, Remark 18 py:F3, lean:F3, leanML:F3
F4	Count-valued comparisons are flag-free: every ratio of two quantities of equal crossing degree ($Gm/r^{3}$, the phase exponent [E][T]/[ℏ]) is a neutral or flag-free label, the flag entering unitful measures only; F/a=[m], crossing degrees 1 and 0, is the flagged boundary case.	T	Examples 5 and 8, Section 7, Remark 17 py:F4, lean:F4, leanML:F4
F5	The electromagnetic domain: with the Coulomb constant neutral (declared), ${\left[q\right]}^{2}=\left[E\right]\left[L\right]=i_{D}\left[L\right]{\left[T\right]}^{-1}$; charge is an integer degree on the lift realised as ${\left[L\right]}^{2\kappa +1}{\left[T\right]}^{(\kappa -1)/2}$, existing iff κ is odd, unique up to $i_{D}^{2}$, never window-covariant (|r|=2κ); no further generator within C1’s two charts; on the even capacities no label squares to ${\left[q\right]}^{2}$.	T	Remark 21 py:F5, lean:F5
**G. Periodicity, windows and the worked examples**			
G1	Local recovery: for an exponent horizon *H* with 4κ>2H the modular labels distinguish every conventional pair |r|,|s|≤H; the flagged sector is recovered through the crossing degree, $M^{u}L^{a}T^{b}\mapsto \left(a-2u,\;b+u;\;u\right)$ injective and windowed-faithful.	T	Theorem 10, Corollary 9, Remark 20 py:G1, lean:G1, leanML:G1
G2	The window ladder: coherence $2\sqrt{\kappa }$ below recovery κ/2 below flag inaccessibility κ below covariance 2κ, nested strictly for every κ≥17 and failing for the toy κ=3, all orderings by integer squares; the coherence identity ${\left(2\sqrt{\kappa }\right)}^{2}=p-1$ and the totality closure $(2\sqrt{{S)}^{2}=\Omega -1.}$	T	Remark 19 py:G2, lean:G2, leanML:G2
G3	The worked examples: κ=3, H=5, 12>10 so every pair in ${\left[-5,5\right]}^{2}$ is distinguished; kinetic energy $\left[m\right]{\left[v\right]}^{2}=\left[E\right]$; $Q+Q^{2}$ inhomogeneous; the phase exponent neutral; flag arithmetic ($G\hbar /c^{3}$ flag-free, $i_{D}^{2}={\left[T\right]}^{\pi }$); the Schwarzschild length $\left[Gm/c^{2}\right]=\left[L\right]$; the gravitational frequency $\left[Gm/r^{3}\right]={\left[T\right]}^{-2}$; the energy–momentum relation $E^{2}=p^{2}c^{2}+m^{2}c^{4}$ homogeneous at crossing degree two, its massless case E=pc at crossing degree one.	T	Exemples 1–8 py:G3, lean:G3, leanML:G3
G4	Buckingham’s count: on the integer lift the neutral monomials of *N* quantities are the kernel of their N×3 label matrix, N−rank independent dimensionless products with rank ≤3, the classical count for quantities whose classical dimensions lie in *M*-*L*-*T* and the derived-$k_{B}$ count for thermal ones; on the window 2H<κ, |j|≤1 lifted and realized neutrality coincide; the pendulum (T,ℓ,g,m): rank 3, one product.	T	Corollary 10 py:G4, leanML:G4
G5	The resolution floor: a registration resolved to one part in *N* is a coherent count of *N*, bounded at $\sqrt{p}$ (A5), so a single coherent registration on a shell of cardinality p resolves no finer than $p^{-1/2}$; under $p^{2}<\Omega$ the floor is at least $\Omega^{-1/4}$ on every admissible shell, of the order of $10^{-31}$ (102 bits) at $S=2.6\times 10^{122}$ (Remark 1), thirteen orders below the $10^{-18}$ optical-clock comparisons and twenty-six below the uncertainty of *G*.	T	Remark 16 py:G5, lean:G5
**P. Predictions**			
P1	The resolution floor, staked: no single coherent registration resolves finer than $\Omega^{-1/4}$, of the order of $10^{-31}$, and no coherent count exceeds $\Omega^{1/4}\approx 6\times 10^{30}$ (G5 over A2, A5, B1; the holographic reading of Remark 1). Falsifier: one coherent registration finer than that. Confrontation: the finest interspecies frequency ratio, $3.2\times 10^{-18}$ [44], and the finest differential comparison, $7.6\times 10^{-21}$ [45]—67 bits against 102, averaged determinations; the mass-independent clock bound ${\left(t_{P}^{2}t\right)}^{1/3}$ permits finer beyond 730 s.	T	Remark 16, Section 9 py:P1, lean:P1
P2	Constancy, staked: every dimensionless ratio of the constants is fixed in time and position, zero drift, zero dipole—the totality timeless (B1), every constant a residue of it in the framework’s realisation (A1; the quartet’s, E1, E6; the charge’s, Remark 21), the quartet’s fixed by the congruences given S (E4), selected once (E7). Falsifier: a detected variation (E1’s). Confrontation: $\dot{\alpha }/\alpha =1.8\left(2.5\right)\times 10^{-19}\;{\mathrm{yr}}^{-1}$ [51], consistent with zero.	T	Section 6 and Section 9 py:P2, lean:P2
**X. What the construction explains**			
X1	The arity of classical bookkeeping, derived: four horizons less one identity, 4−1=3 free scales in bijection with {c,ℏ,G}, exactly three independent members of $\left\{c,\hbar ,G,k_{B}\right\}$; the three mechanical primitives are the two chart generators and the flag’s torsion-free surrogate, mass, $\left[m\right]=i_{D}{\left[L\right]}^{-2}\left[T\right]$, $m_{P}=p_{P}/c$. Classical dimensional analysis postulates the count and the mass primitive.	T	Corollaries 4 and 5, Theorem 8, Properties 10 (restates E2, E3, F2) py:X1, lean:X1, leanML:X1
X2	The thermal primitive is redundant: $\left[\Theta \right]=\left[E\right]{\left[k_{B}\right]}^{-1}=\left[L\right]{\left[T\right]}^{-2}$, the acceleration domain, flag-free where energy carries the flag; $|k_{B}|=\hbar /c$ is the quartet’s mixed product; the Unruh combination $\hbar a/(c\;k_{B})$ closes as a domain identity. Classical bookkeeping adjoins a thermal unit and reads $k_{B}$ as a conversion.	T	Corollaries 3 and 7, Remark 18 (restates E3, F3) py:X2, lean:X2, leanML:X2
X3	One action quantum for both conjugate pairs: the domain lattice has exactly one order-four subgroup, in the time factor (no flag of space), canonical and covariant, so E=hf and p=ℏk share one *ℏ*, $\ell_{P}p_{P}=t_{P}E_{P}$—derived from the shell’s algebra with no Lorentz structure used.	T	Theorem 7, Corollary 1, Properties 5, Remark 11 (restates D2, E2) py:X3, lean:X3, leanML:X3
X4	Charge is an integer degree of the two generators on the shells of odd capacity, not a further primitive: with the Coulomb constant neutral (F5), ${\left[q\right]}^{2}=i_{D}\left[L\right]{\left[T\right]}^{-1}$, $q_{P}^{2}=\hbar c$; the lift has no half root (the Gaussian exponents $M^{1/2}L^{3/2}T^{-1}$ are half-integers there), so torsion-free bookkeeping adjoins an electrical primitive as it adjoins mass; the fine-structure ratio stays a neutral count.	T	Remark 21 (restates F5) py:X4, lean:X4

Collecting the tags separates what a reader must grant from what is derived. *Imported* are the framed substrate and the realisation doctrine (A1), the holographic count (A2), the shell Fourier structure (A3), classical dimensional analysis and the Planck units (A4), the registrability bound (A5) and the Unruh form (A6). *Declared* are the Carrier and the Subject (B1), the Object (B2), units as scale assignments (B3), the phase and framed-complex labels (B4), the domain generators and the quantity algebra (C1), the admissible reframings (C4), the space-exponent refinement (the declared half of C6), the crossing degree (D4), the residue assignments (the declared half of E6), the two moves (E8) and the Coulomb constant neutral (the declared half of F5). *Realised* are four rows, each with its falsifier: the unit flag on the Planck constants (D5), the unit as a reciprocal relation (D7), the quartet as the Carrier’s horizons (E1) and the horizon declarations (the realised half of E6). *Predicted* are the two rows of block P, each a theorem consumed through the realisations it names, tagged T | R, with its falsifier and its present confrontation: the resolution floor (P1, over A2, A5, B1 and the holographic reading of Remark 1) and the constancy of the dimensionless ratios (P2, over A1, B1, E1, E4, E6, E7). *Theorems* are the twenty rows of blocks C–G tagged T (with the forced half of C6 and the theorem half of F5): the domain algebra (C2, C3, C5, the forced half of C6), the four domains and the flag (D1–D3, D6), the quartet’s identities and its congruences (E2–E5, E9), mechanics and temperature (F2–F4, the theorem half of F5), recovery, the ladder, the examples and the resolution floor (G1–G5); every one of them exact, the witnessed ones decided by the package on the named shells and Carriers (G5 and P1 on the chart value, P2 on the imported bound). Of these, C2, C3, C5, C6, D1–D3, D6, E2–E5, F2–F4, G1 and G2 are proved in Lean on Mathlib’s hierarchy for every shell and Carrier, C2, D1, D2, D6, E4, E5 and P2 in the core with no axioms, E9, G3, the roundings of G5 and P1, the two Carriers and F5’s charge labels (their two values on κ=3,7, their absence on κ=4,10) are decided by the kernel, and G4 is proved for the pendulum on the integer lift (its kernel the line of $T^{2}g/\ell$, a nonzero minor) with the window coincidence, on the neutrality criterion of C3 and the embedding of G1; the count for general *N* and the parity criterion of F5 for every κ are not formalised. Block X restates six of these theorems in four rows (X1–X4) as what classical bookkeeping postulates and the construction derives—the arity and the mass primitive, the thermal primitive, the single action quantum, the electrical primitive; its rows add no theorem and carry the witnesses of the rows they restate. Ω*-hard* is the Carrier register itself: the absolute unit values and the member selection of each residue pair (E7). No row is open. The paper’s title claim—dimensional analysis as the finite shell-domain calculus with the unit flag—is C2 with D2, carried to the constants by E1, E2 and E4.
